# Protein Dynamics in F-like Bacterial Conjugation

**DOI:** 10.3390/biomedicines8090362

**Published:** 2020-09-19

**Authors:** Nicholas Bragagnolo, Christina Rodriguez, Naveed Samari-Kermani, Alice Fours, Mahboubeh Korouzhdehi, Rachel Lysenko, Gerald F. Audette

**Affiliations:** 1 Department of Chemistry and the Centre for Research on Biomolecular Interactions, York University, Toronto, ON M3J 1P3, Canada; nickb17@yorku.ca (N.B.); csr07@yorku.ca (C.R.); natyork@my.yorku.ca (N.S.-K.); 2 Department of Chemistry, York University, Toronto, ON M3J 1P3, Canada; alice4s@my.yorku.ca (A.F.); mahboubeh.v90@gmail.com (M.K.); lysenko.rachel@gmail.com (R.L.)

**Keywords:** bacterial conjugation, type IV secretion system (T4SS), F plasmid, pilus protein structure, protein dynamics, protein-protein interactions, targeted drug design

## Abstract

Efficient in silico development of novel antibiotics requires high-resolution, dynamic models of drug targets. As conjugation is considered the prominent contributor to the spread of antibiotic resistance genes, targeted drug design to disrupt vital components of conjugative systems has been proposed to lessen the proliferation of bacterial antibiotic resistance. Advancements in structural imaging techniques of large macromolecular complexes has accelerated the discovery of novel protein-protein interactions in bacterial type IV secretion systems (T4SS). The known structural information regarding the F-like T4SS components and complexes has been summarized in the following review, revealing a complex network of protein-protein interactions involving domains with varying degrees of disorder. Structural predictions were performed to provide insight on the dynamicity of proteins within the F plasmid conjugative system that lack structural information.

## 1. Introduction

Bacterial conjugation is a method of horizontal gene transfer considered to prominently contribute to the propagation of virulence genes among pathogens [1,2,3]. Conjugative plasmids are transmissible through the type IV secretion systems (T4SS) encoded within a transfer (*tra*) or virulence (*vir*) operon, which allows the plasmid carrier to mediate donor-to-recipient DNA transfer [4]. As plasmids often undergo transposition events with the bacterial chromosome, integrative, and conjugative elements increase the rate of bacterial evolution by enhancing the plasticity of their genomes [5]. The misuse of antibiotics in animal agriculture and over-prescription of broad-spectrum antibiotics has resulted in the selective evolution of antibiotic resistance genes, which commonly migrate onto conjugative plasmids [6,7]. As conjugative plasmids are ubiquitous in the bacterial kingdom, inhibition of conjugative T4SS has been suggested as a drug-design target to prevent the dissemination of antibiotic resistance genes; thus, preventing the proliferation of multi-drug resistant pathogens [8,9,10].

**Figure 1:**
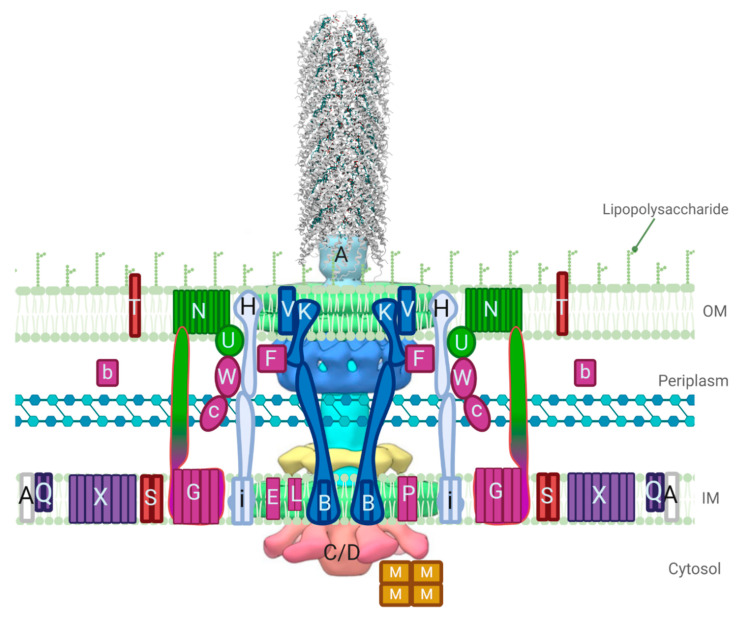
A model of the F-like type IV secretion systems (T4SS) based on available structural information, including a cryo-electron tomography (cryo-ET) model of the pED208 core complex and a cryo-electron microscopy (cryo-EM) model of the assembled pED208 pilus (PDB ID: 5LEG) [15,16]. Tra proteins are labeled with capital letters and Trb proteins are shown with lower case letters. Proteins are colored based on function; the pilin TraA is white, pilin processing proteins are purple, proteins responsible for pilus assembly/extension are colored in fuchsia, the core complex is colored in dark blue, while pilus retraction proteins are colored in light blue, green colored proteins are responsible for mating pair stabilization, and the red proteins are responsible for exclusion. ATPase proteins TraC and TraD are complexed in the cytosol based on the cryo-ET image, but are present in the inner membrane (IM) as well. The outer membrane (OM) complex in blue is composed of TraK and TraV in a complex that creates 13-fold symmetry. Figure created with BioRender.com.

The process of bacterial conjugation is complex, and the mechanisms of the many stages involved in the transfer of conjugative DNA from a donor to a recipient cell are poorly understood as they vary in different plasmid families [3,11]. Plasmids code for the genes required in the assembly of a T4SS and the transfer of the plasmid DNA; in incompatibility (Inc)F-type plasmids these genes are located in the *tra* operon, and expression is regulated by proteins and RNA upstream and within the operon [12,13]. Once the Tra and Trb proteins from the T4SS are expressed and assembled, pilin processing events occur, followed by pilin assembly and extension (Figure 1) [14]. After a recipient cell has been contacted, pilus retraction proceeds, and in some systems, such as the F plasmid, this is followed by a mating pair stabilization step which allows for the close contact of cell membranes. Exclusion events then occur to prevent redundant transfer of conjugative DNA to a recipient cell that has the same plasmid. A mating bridge is then formed between the cells to allow for the transfer of the plasmid by a Tra protein complex called the transferosome, thus, allowing rolling circle replication to occur.

There is evidence of a high degree of structural dynamicity in multi-protein complexes that span the bacterial cellular envelope. For instance, type 4 pili, which are generated by type 2 secretion systems, function in motility, natural competence, phage adsorption, protein secretion, and surface sensing and have been shown to feature disorder throughout their structures [17]. In analyzing the available structural information of the components of the conjugative F-like T4SS, such as protein structures deposited in the Protein Data Bank (PDB), a similar diverse structural ensemble is seen where proteins that have high structural integrity, and are well conserved, are seen to interact with proteins that display various levels of disorder. There is some evidence that dynamic proteins, and their sites of interaction with other proteins in a given protein complex, can be optimal drug targets if the structural and conformational dynamics of the protein are understood [18,19]. The following review summarizes what is currently known regarding the structural dynamics of F-like conjugative T4SS components, and highlights tactful targets for rational drug design. In addition, a Phyre2 [20] analysis of all known Tra and Trb proteins was performed to predict the disorder present in proteins with unknown structure and to estimate the novelty of their folds.

## 2. Dynamics of Proteins Involved in the Regulation of Bacterial Conjugation

The transcription of genes coding for conjugative T4SSs is strictly regulated as its production and subsequent operations are metabolically costly processes for bacterial cells [21,22]. The expression of conjugative systems is controlled by either the basal level of constitutive gene expression, or signaling molecules (such as autoinducers or sex pheromones used in quorum sensing), or both [23]. The regulation of conjugation in plasmids from the IncF family of gram-negative bacteria occurs solely by the former process and has been termed fertility inhibition (fin) [24]. In the canonical F-plasmid, the gene coding for the activator of the transfer (*tra*) operon expression, *traJ*, is controlled by *finOP* regulatory genes. TraJ expression is downregulated by the binding of *traJ* transcripts with antisense RNA FinP. The protein FinO binds and protects FinP RNA from RNase E degradation, and promotes duplex formation between FinP and *traJ* RNA in a predicted kissing motif [24,25,26,27,28,29,30]. This antisense RNA control mechanism reduces F transfer 10–2000 fold [31]. Many plasmids from the F-like family have an insertion sequence 3 (IS3) element in the FinO gene and low constitutive expression of FinP; therefore, expression of TraJ is not inhibited, resulting in high constitutive expression of the *tra* operon [24].

While these proteins are not components of the T4SS, their function is intimately linked to the regulated production of their respective conjugative system [24,25,26,27,28,29]. Therefore, novel antibiotics could be developed to interact with these proteins, transcripts, or genes to prevent or overexpress the production of the T4SS. Such interactions could result in the reduction of the spread of virulence genes by decreasing the expression of conjugative T4SS proteins or cause cell death through overproduction of the large macromolecular complex [21,22].

### 2.1. FinO and FinP

**Figure 2:**
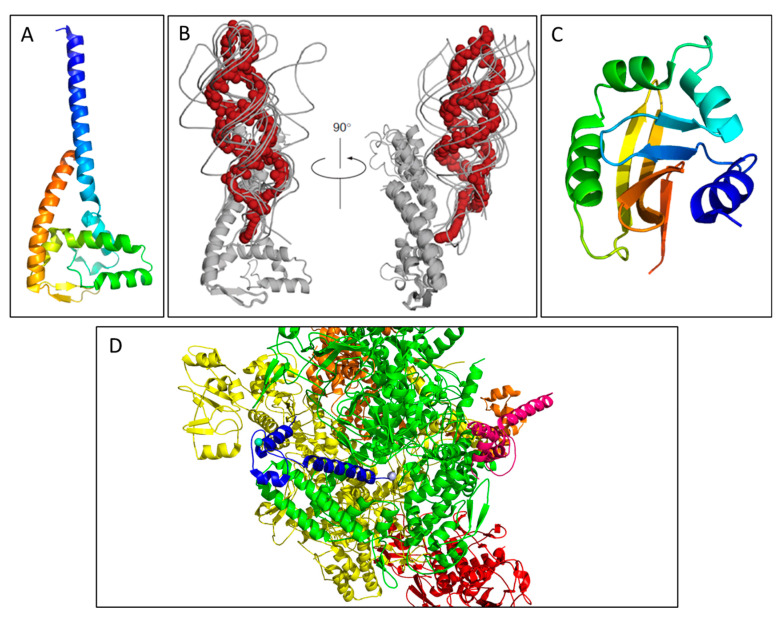
Structures of proteins with transcription regulatory roles in the F-plasmid *tra* operon. (**A**) The crystal structure of a truncated FinO lacking the disordered N-terminal domain (PDB ID: 1DVO). This structure was used for modeling of small angle X-ray scattering (SAXS) data shown in (**B**), which displays the dynamic conformational ensemble involved in the binding of FinP SLII RNA (red spheres display the average structure of the best cluster of nine complexes, also shown as grey ribbons) to a truncated FinO monomer shown as a grey cartoon [28]. (**C**) The structurally conserved Per-ARNT-Sim (PAS) domain of TraJ; it is the only region of a TraJ structure currently solved to high resolution (PDB ID: 4KQD). (**D**) The structure of TraR bound to RNA polymerase (RNAP) in the conformation exhibiting the highest occupancy as determined by cryo-EM. TraR, shown in blue, is seen with its N-terminal helix extending from the secondary channel of the active site in the β-subunit colored green. These residues are shown to interact strongly with the RNAP motif that chelates the Mg^2+^ ion seen in grey (PDB ID: 6N57). Structural images in Figure 2A–D, Figures 4 and 5 were generated with PyMOL [35]. Figure 2B was adapted with permission from Arthur et al. Mapping interactions between the RNA chaperone FinO and its RNA targets. *Nucl. Acid Res.* **2011**, *39*, 4450-63 [28].

The RNA binding protein FinO features a disordered N-terminal region from Met1-Lys25 that prevented the protein’s crystallization, the remainder of the 186 residue protein from the F-like plasmid R6-5 was crystallized and solved by X-ray diffraction (Figure 2A) [25]. The structure of FinO is highly elongated and mainly α-helical, with flexible N- and C-terminal helices extending from a well-structured central region composed of five helices and two β-hairpins. The terminal helices are highly positively charged and have been shown to bind RNA through proteolytic assays [28,29,30,32]. The stem loop II (SLII) region of FinP and its 3′ nucleotides have been shown to bind these respective surfaces with high affinity to increase the stability of the transcript. As well, it is predicted that this positions the RNA, such that *traJ* RNA can be bound in a predicted kissing complex [28]. While no crystal structures of a FinO-RNA complex have been solved, a low-resolution model of the FinO-SLII complex has been presented using small angle X-ray scattering (SAXS) and corroborated with various assays (Figure 2B) [28].

The flexible N-terminus that was removed for proper crystallization was determined to be functional in promoting strand exchange of the RNA with *traJ* RNA transcripts through uncoiling stem loops [29,30]. The α_1_ helix has been shown to be especially important in promoting strand exchange as it features a positively charged surface and many key hydrophobic residues, as seen through point mutation assays. The presence of disordered regions in RNA chaperone proteins is common and not mere coincidence for FinO; while the N-terminal region appears to be dispensable for the in vivo stability of FinP, deletions of the region strongly reduce the ability of FinO to repress conjugation [29,33,34]. FinO_45–186_ exhibits no inhibition of conjugation, even though this N-terminal truncation mutant was demonstrated to protect FinP against degradation similarly to the full-length protein [29]. This demonstrates the often-overlooked importance of highly dynamic regions; targeting this region with an antibiotic could result in the uncontrollable expression of an F-like T4SS that would greatly encumber bacterial cellular processes.

### 2.2. TraJ

The transcriptional activator of the *tra* operon, TraJ, functions through the binding of promoter P_Y_ as a homodimer [36,37,38,39]. Interaction of TraJ with the promoter helps to relieve the binding of P_Y_ DNA to histone-like nucleoid structuring proteins (H-NS) to enable binding of transcription factors [40,41]. TraJ also interacts with host factor protein ArcA to aid in the transcriptional activation of the *tra* operon by an independent mechanism [42,43].

Crystal structures of the N-terminal domain (NTD) of TraJ from plasmids F and pSLT have been shown to feature a Per-ARNT-Sim (PAS) fold, a common sensor motif seen in signaling proteins (Figure 2C) [41,44,45]. Despite dissimilar sequence homology in this domain, the structural homology of the fold is highly conserved [41]. All F-like TraJ proteins are predicted to have a PAS motif in their NTD, which has been shown to be responsible for the homodimerization of TraJ [38,41,46]. An NTD swapping experiment of TraJ from F-like plasmids resulted in similar levels of activation in hybrids compared to the native TraJ proteins, indicating functional interchangeability of the domain containing the PAS fold [41].

The structure of the remaining C-terminal residues of TraJ has not been determined in high resolution; however, there is a known helix-turn-helix (HTH) motif present in all F-like TraJ homologs that has been determined to be responsible for P_Y_ DNA recognition [38,41,46]. Predictably due to the HTH motif, the C-terminal domain (CTD) of TraJ displays functional specificity, as lowered activation of the *tra* operon was seen when TraJ is interacted with the P_Y_ from a non-cognate F-like plasmid [41,47]. It is predicted that the dimerization of TraJ is required for its proper folding, indicating the dynamic nature of TraJ in achieving its active conformation only through dimerization [41].

### 2.3. TraR

The role of the transcription factor TraR in the process of conjugation is unconfirmed as the protein is dispensable for plasmid transfer; however, it is known to bind RNA polymerase (RNAP) to affect gene transcription [48,49,50,51,52]. TraR is in the class of proteins that binds the secondary channel of RNAP, which results in transcription inhibition of ribosomal RNA (rRNA) genes and activates transcription of amino acid (aa) biosynthesis genes [52,53]. DskA is the best studied member of this class as it is a highly conserved protein in proteobacteria; DskA only binds RNAP in a manner similar to TraR when in the presence of guanosine tetraphosphate (ppGpp), a cell stress-related nucleotide [49,52]. Speculation has been made to whether TraR confers indirect fitness advantages to the host, thus increasing the survivability of the plasmid [49,50]. DskA and ppGpp have been shown to modulate auto-aggregation and motility genes; both functions can lead to new successful conjugation events [52]. Genes found on pathogenicity islands are also upregulated by the presence of DskA and ppGpp, and, therefore, TraR is likely to increase production of these virulence genes as well. As ppGpp is not required for TraR activity, gene upregulation normally associated with stress-response occurs without the associated environmental stressors. The function of F TraR may be masked by constitutive expression of the F T4SS due to the mutation of *finO*, or perhaps it plays a more notable regulatory role in conjugation when the natural conditions of a competitive microenvironment are present [49,50].

A notable structural feature of TraR is its small size at 73 aa, and when compared to DskA it shares 29% sequence homology, all of which lies in the C-terminal region [52]. Structures of TraR bound to RNAP show that the N-terminal α_1_ helix is analogous to the 2nd helix of the DskA coiled-coil domain, both of which fit into the secondary channel of RNAP [48,54]. Conserved TraR residues Asp6 and Ala8 have analogous residues in DskA; Asp6 binds the nucleotide triphosphate (NTP) binding site, and Ala8 fits into the cavity of the bridge helix, and when mutated, function is severely impaired [48,49,54]. The N-terminal helix of TraR binds the same residues of RNAP as the coiled-coil domain of DskA when ppGpp is also bound; however, the conformation of TraR allows for more direct interaction with RNAP (Figure 2D) [48,54]. The hydrophobic residues Ile23 and Ile27 of TraR occupy the space filled by ppGpp; thus, providing a structural justification for the activity of TraR on RNAP function despite a lack of signaling nucleotide. TraR is a well-structured protein and induces a conformational change upon binding to RNAP; however, three major conformational states of TraR bound to the RNAP have been discovered by cryo-EM with a continuous distribution of conformations between them, indicating the inherent flexibility of the small protein when bound to its target [54].

## 3. Structures Involved in Pilin Processing, Pilus Extension, and Retraction

### 3.1. Proteins Responsible for Propilin Maturation

The conjugative pili, an external appendage ubiquitous in the bacterial kingdom, mediates bacterial conjugation. On average, an *Escherichia coli* cell will have anywhere from 1–5 pili, with some variants having up to 20 [55]. Pili can range in length and flexibility; cells expressing rigid pili use these appendages to manifest an aggregate network of rigid polymers, thus, encouraging non-specific clumping of donor and recipient cells to form mating pairs [56]. Some pili, such as the F pilus, are capable of extension and retraction allowing for close contact between a donor and recipient cell for bacterial conjugation. Other pili will use retraction and extension as a manner of maneuverability termed “twitching motility” [57]. During phage infection, prior to DNA or RNA being injected into a bacterial cell, a major site of absorption is the pilus [16,58]. As the pilus is an important aspect in virulence, as well as providing bacteria adaptivity to their environment it, is deemed an attractive target for novel therapeutics. 

The first step in pilus production is the post-translational processing of propilin subunits. Within the F *tra* system, proton motive force embeds TraA within the IM in a sec-independent mechanism [59,60]. The inner membrane (IM) protein TraQ then interacts with proTraA to ensure stabilization within the membrane. ProTraA is synthesized with a leader sequence; this sequence is acetylated by TraX at A52 and the preceding N-terminus is cleaved to produce the mature pilin [61]. Mature TraA is then extracted from the IM for pilus assembly.

#### 3.1.1. The Pilin Protein TraA

**Figure 3:**
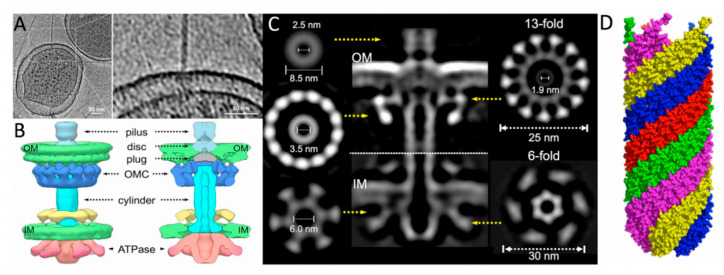
The F-like pilus and T4SS. Figures **A**–**C** are in situ structures of the F-like pED208 T4SS by cryo-ET while (**D**) depicts the overall architecture of the pED208 pili by cryo-EM. (**A**) A tomographic slice of an *E. coli* minicell showing an extended F pilus attached to the OM. (**B**) A three-dimensional depiction of the pED208 core T4SS from ATPase to pilus. At the OM, the pilus narrows and connects to the disc at the junction where the pilus meets the OM complex; a luminal space approximately the same diameter of the pilus is present at this junction, which is sealed by a plug domain. (**C**) A central slice of the averaged structure of the core T4SS with cross-sectional views of the separate domains. The IM complex (the VirB4-like ATPase, TraC) forms a 6-fold rotational domain with a central lumen of ~60 Å with the entire domain spanning 300 Å. The OM complex (the VirB7, -B9, and -B10 homologues TraV, TraK, and TraB) has a 13-fold symmetry and spans 250 Å with a central lumen of 19 Å. (**D**) A side view of the surface representation of the pED208 pilus (PDB ID: 5LEG). It is composed a of a 5-start helical assembly with each helical strand depicted in a separate color. This cryo-EM structure aligns with the architecture of the cryo-ET pilus structure in that it spans ~87 Å and has a central lumen of 28 Å. Figure 3**A**–**C** was adapted from Hu et al. Structural bases for F plasmid conjugation and F pilus biogenesis in *Escherichia coli*. *Proc. Natl. Acad. Sci. USA* **2019**, 201904428 [15].

The propilin protein, TraA is a small protein ~13 kDa that is processed by the leader peptidase LepB to a much smaller ~7kDa protein [58,59]. The *traA* gene is composed of 121 amino acids with a signal peptidase (SP) cleavage site present between Ala51 and Ala52, TraA is processed to a mature pilin of 70 amino acids represented by residues Ala52–Leu121 [62]. The propilin is then further processed by proteins TraQ and TraX. Structures of the assembled pili composed of mature TraA_F_ and TraA_pED208_ have been resolved by cryo-EM with the latter having higher resolution [16]. Both TraA proteins have four α-helical domains which form similar all α-helical structures [16,37]. Both the NTD and the CTD are on the exterior of the assembly, with domain I exposed as well, domains II and IV are hydrophobic and span the IM prior to assembly with domain III extending into the cytoplasm to connect them, and a basic loop between domains II and III is exposed to the cytoplasm [11,63]. These structural localizations agree with the proposed mechanism of phage attachment to TraA, which is predicted to be mediated through association with the NTD and CTD of TraA, and also promotes the hypothesis that DNA may pass through the pilus, as the pilus architecture allows for an inner lumen of 19 Å that may allow for the transferosome to be chaperoned; the single-stranded DNA (ssDNA) would be stabilized by the positively charged loop created between domain II and III (Figure 3D) [16,55].

#### 3.1.2. TraQ

The stabilizing protein TraQ is thought to be a bitopic IM protein that interacts with TraA for rapid insertion of the pilin protein into the IM [64]. The *traQ* gene encodes a 94 aa protein with the majority of the C-terminal end containing charged or polar amino acids and, as such, is thought to be oriented with the N-terminal end facing the cytoplasm and the C-terminal end facing the periplasm [65,66]. TraQ is required for propilin processing to achieve its correct orientation and stabilization [55]. It is predicted that TraQ is capable of binding specifically and directly to the TraA domain IV for proper membrane insertion [65]. This interaction is transient so that TraQ is free to participate in multiple cycles of binding propilins and stably incorporating them within the membrane. In the absence of TraQ, TraA exhibits a conformation that affects proper membrane translocation and results in degradation of the misfolded TraA by cytoplasmic enzymes [58,64].

#### 3.1.3. TraX

Propilin modification by N-terminal acetylation is achieved by the expression of the *traX* gene. The *traX* gene encodes a mainly hydrophobic IM protein of 248 aa that has also been detected to produce two small products in all observed F-like plasmids; TraX1 and TraX2 [8,14]. TraX1 and TraX2 are 22 and 24 kDa, respectively, and end with the same C-terminal sequence; however, their function is unknown. Though TraX is required for the acetylation of propilin, TraX is not required for pilus biogenesis [67]. The structure of TraX has yet to be solved and is difficult to predict, likely due to the instability of N-acetylases following purification and the rarity of bacterial N-acetylases [67,68]. Structural analysis by Phyre2 predict that TraX is a polytopic protein with eight transmembrane (TM) domains; however, earlier predictions estimated TraX has four TM domains [20,37,69].

### 3.2. Pilus Assembly and Extension Proteins

F pili extension/retraction is different from non-conjugative type IV pili, the best studied models of the type II secretion systems, in several key regards. Fluorescence microscopy imaging has shown F pili rotate throughout the process of pilus extension while type IV pili do not, indicating that the molecular motors involved in the F pilus remain stationary leading to pili rotation [70,71]. Furthermore, type IV pili encode two different hexameric adenosine triphosphatases (ATPases), PilF (for extension) and PilT (for retraction), in contrast to the F-like T4SS family in which retraction is considered to occur in an energy-independent manner; TraC and TraD are proposed to supply energy to the system for pilus extension and transferosome transport cooperatively [15,71].

This intricate process of assembling TraA pilin monomers requires the aid of many proteins, including TraB, -C, -E, -F, -G, -H, -K, -L, -U, -V, -W, and TrbC [11]. Of these proteins, TraF, -H, -U, and -W are specific to the F T4SS, all others have structural and functional homologs [14]. Mutations in these *tra* and *trb* genes cause various disruptions in the assembly of the pilus, from alterations in length, to completely inhibiting the formation of a pilus. Although these proteins are known to be involved in the assembly and/or extension of the pilus, the structure and function of each is largely unknown.

The pilus is composed of mature TraA subunits, which assemble into a helical structure with a length of approximately 20 μm, an outer diameter of 8–10 nm, and an inner lumen of 2–3 nm in diameter [11,72]. The structure of the pilus has been determined by scanning transmission electron microscopy (STEM), where the subunits were found to be mainly α-helical and the pilus exhibited a 5-fold rotational symmetry [58,73]. A separate cryo-EM structure provided a higher resolution image of the five-start helical structure composed of ~12.8 TraA pilins per helical turn (Figure 3) [16].

#### 3.2.1. Pilus Tip Formation

Five proteins are involved in the formation of the pilus tip: TraE, -C, -G, -K and -L. TraE is a mainly hydrophobic protein found within the IM, thought to be involved in the process of pilus tip formation as well as the assembly of the IM complex [37,55]. Mutations in *traE* result in the prevention of pilus tip formation [14]. TraE has been purified from the complex in sizes from 130–261 aa, but is predominantly found as a 188 aa protein [14]. It is thought that TraE may contain a leader sequence that is cleaved prior to insertion into the IM [37,62]. This predominantly hydrophobic protein forms an N-terminal membrane anchor from residues Val12−Ile33 [37]. According to Phyre2, the structure of TraE is thought to span the membrane a single time and is composed of mainly α-helices in the N-terminal region and β-strands in the C-terminal region, with a low amount of disorder throughout [20]. TraL is a mainly hydrophobic protein localized within the IM thought to limit the number of F pili produced by a cell [14,37,74]. The TraL family of proteins range in size from 93 to 105 aa with a shared homology to VirB3 from the Ti plasmid and TrbD from the RP4 plasmid [75,76]. TraL from the F plasmid is 91 aa and is predicted to localize within the IM [77]. The predicted structure of TraL contains two TM α-helices bracketed by reverse turns, followed by regions of β-structure connected by a reverse turn [62]. TraC will be discussed in further detail as its ATPase activity is crucial for providing energy to the T4SS for pilus extension. The NTD of TraG is embedded within the IM and has an unknown role in the assembly of the pilus tip. It is predicted to have three-to-five TM-spanning helices in the first 445 aa with the remaining CTD as a periplasmic region participating in other conjugative roles [37,78,79]. TraK is a predominantly hydrophilic protein that is localized in the periplasm, which is thought to interact with the lipoprotein TraV to form the envelope-spanning core complex into which the assembled pilus is extended [80]. Both proteins were shown to be involved in the initiation of pilus formation through M13K07 phage sensitivity assays of protein mutants [81].

#### 3.2.2. T4SS Core Proteins TraB, -K, and -V

TraB from F-like plasmids range between 429 to 475 aa and share homology in their CTDs with TrbI from IncP plasmids (TrbI_P_), VirB10_Ti_, and TraO_I_ [14]. TraB from the F plasmid is 475 aa with a small N-terminal IM-bound domain and the remaining protein extended into the periplasm where it forms an envelope-spanning complex with the mainly periplasmic TraK and TraV in the OM [80]. Utilizing yeast two-hybrid analysis, TraB residues Met138−Asp229 were shown to bind the region Thr6−Ala27 in TraK found in the periplasm, which interacts with the TraV residues Arg127−Asn171 in the OM using the domain Thr124−Arg237. It is hypothesized that TraK serves as a secretin to aid in creating an envelope structure spanning across the membrane allowing for pilus extension through the bacterial cell envelope [14]. The pED208 core complex solved by cryo-ET includes homologs of these proteins in the OM complex; despite the low resolution, three-dimensional classifications revealed the complex displays 13-fold symmetry (Figure 3A–C) [15]. TraB, -K, and -V are essential for F plasmid conjugation; therefore, the sequences of interaction between the proteins composing the core complex could be presented as useful targets for drug discovery.

TraB is a predominantly hydrophilic protein anchored in the IM by Ile13-Leu33 [76]. The region in TraB predicted to be responsible for TraK interaction, Met138-Asp229, was found to be proline-rich at 23% of the residues in the segment [80]. Proline-rich segments are common in eukaryotic cell protein–protein interactions (PPIs); however, it is not known whether a proline-rich binding motif mediates the interaction between TraB and TraK. Structural predictions using Phyre2 show a high degree of disorder in the protein at 44%, but did not accurately estimate the location of the IM-bound region; highest confidence models were found in the periplasmic CTD exclusively [20].

The TraK family of proteins are homologous to TrbG_P_, VirB9_Ti_ and TraN_I_ and range between 299–410 aa [37]. The TraK family shares similarities to secretin proteins, particularly the HrcC subgroup of the type III secretion system (T3SS) encoded by *Pseudomonas syringae* [82]. The β-domain and S-domain of TraK is identical to the secretin PulD of *Klebsiella oxytoca* [83]. The β-domain is theorized to be embedded in the OM, and form a ring structure typical of most secretins. The S-domain consists of a 60 aa sequence that binds to a lipoprotein acting as a periplasmic chaperone [84]. The C-terminus interacts with TraV while the N-terminus interacts with TraB, and it is predicted that TraK could act as a chaperone for TraV [80]. Estimations of secondary structure characteristics by Phyre2 show the protein displays a mixed α−β structure with a predicted disorder content of 34% [20].

TraV is a lipoprotein with a conserved cysteine at the N-terminal signal sequence, with sizes ranging between 171 to 316 aa in the F-like plasmid family and is 171 aa in the F plasmid [80]. There are two conserved cysteines that appear to allow for multimerization and/or interaction with TraK [85]. TraV is predicted to be an OM anchor for the TraV−K−B complex [80]. Phyre2 predicts the structure of TraV to be highly disordered at 61% of the overall secondary structure; however, this value is likely caused by the lack of structural data on small TM proteins [20].

#### 3.2.3. Pilus Extension—TraF, TrbB, TraP, TraC, TraW, and TrbC

**Figure 4:**
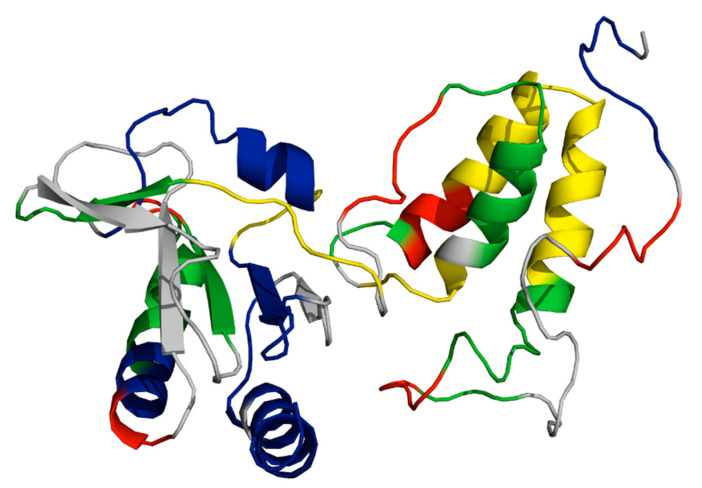
A predicted model of TraF, known to be required for F pilus extension beyond the OM. The model is colored based on observed rates of hydrogen-deuterium exchange (HDX) of main-chain amide hydrogens by time resolved HDX mass spectrometry [86]. Rates of exchange are colored as: red = high, yellow = high−moderate, green = moderate−low, blue = low. The C-terminal domain (CTD) (left side of the figure) contains a thioredoxin-like motif and exhibits low HDX, indicating a well-structured domain. The N-terminal domain (NTD), predicted to be responsible for interaction with TraH, is much more dynamic, as exhibited by increased HDX. Further analysis is required to pinpoint structural features responsible for F pilus extension and examine the changes in protein structure and dynamics upon TraF’s interaction with TraH.

TraF is a 26 kDa periplasmic protein necessary for F pilus extension, and deletions at either termini abolish F pili formation eliminating conjugative gene transfer [86]. The NTD of TraF exhibits increased structural dynamics and is responsible for interactions with TraH; the CTD (Glu127-Leu247) contains a thioredoxin-like fold (Figure 4). Through Phyre2 modeling the structure of TraF is predicted to have at least three α-helices bordered by a four-strand β-sheet [20]. It is hypothesized that both TraF and TrbB proteins have chaperone capabilities based on the conserved thioredoxin-like region [86]. TraF has been shown to directly bind to TraH; if this interaction is inhibited, pilus extension is abolished. Furthermore, TraF is considered to have further vital functions due to the abolishment of conjugative function after mutations in the C-terminal domain; this may be due to the predicted complex formation between periplasmic proteins TraF, -H, -W, TrbB, and TrbC, and potentially, TraU. Of these proteins, TraF, -H, -U, and -W appear to associate with the OM when in the context of the complete transfer apparatus [87]. An interaction between TraF and TraV is hypothesized to localize the periplasmic complex with the core complex; if proven, the point of contact would provide a critical site of T4SS association and therefore be a useful drug target [87].

TraF is homologous to another periplasmic protein from the tra operon of the F plasmid TrbB, however TrbB contains the C-X-X-C thioredoxin motif while TraF displays a thioredoxin-like fold but does not have the C-X-X-C motif [88]. TrbB is a 161 aa periplasmic protein that has been shown to act as a disulfide isomerase, and is assumed to help mediate pilus extension as a protein chaperone [89,90]. The structure of TrbB is predicted to be unique amongst disulfide bond isomerases; the N-terminal domain of TrbB is predicted to be largely unstructured (similar to TraF) and is required for proper function, however it does not act as a dimerization domain [89]. TrbB also requires the host protein DsbD to maintain its active, reduced state. The enzymatic activity of TrbB activity is lower for common redox substrates when compared to that of the host disulfide isomerase DsbC; this lowered activity is considered to enable substrate specificity for interacting partners of TrbB. Neither TraF nor TrbB from F-like T4SS have solved structures, which would aid in understanding their unique functions.

TraP is a 21.5 kDa protein predicted to have both termini in the cytoplasm with a periplasmic loop flanked by TM regions from Leu25−Trp49 and Leu118−Pro141 in the IM [76]. TraP is thought to be an accessory protein, which enhances stability of the TM complex involved in pilus polymerization, as mutations in *traP* seem to have minimal negative effect on pilus extension; the protein is not required for F-conjugation in *E. coli*. The disorder content of the protein’s secondary structure is predicted to be 24% by Phyre2 [20].

ATPases are essential components in T4SS as they supply energy for various processes in conjugation, namely pilus extension and transportation of the transferosome. Due to its role as one of two ATPases of the F-like T4SS (the other being TraD), TraC is considered to be the characteristic protein for mating pair formation (Mpf); the *traC* gene has been used for the classification of F-like plasmids [13]. TraC is an 875 aa protein that is expressed cytoplasmically; however, it is chaperoned to the IM through interactions with other IM T4SS components, specifically TraB, -E, and -L [14,91,92]. If over-expressed TraC forms inclusion bodies with reversible solubilization, which can be indicative of the presence of either intrinsic disordered regions (IDRs) or β-strands that allow for the assembly of amyloid-like fibrils [91,93,94]. The structure of TraC from the F plasmid has not been solved, however Phyre2 analysis shows TraC has high sequence homology to VirB4/TrwB/TraB ATPases which also share structural homology to TraD [20]. Phyre2 also predicts a mixed α-β secondary structure which aligns confidently with the crystal structure of the CTD of VirB4 from *Thermoanaerobacter pseudethanolicus* solved to 2.45 Å [95]. This structure features a RecA-like α/β domain that contains the ATP-binding site and a four-helix bundle domain; a 17 Å negative stained EM model of the core T4SS from pKM101 (of the IncN family) featuring the intact VirB4 homolog TraB displays the NTD binding to the side of the IM core complex, rather than stacking underneath. Cryo-ET models of the core complex from the F-like T4SS pED208 features TraC; it also assembles to the sides of the core complex as a hexamer of dimers, maintaining the active structure seen in the cytoplasm and when complexed in the IM (Figure 3B) [15]. The structure of TraC_F_ is likely most similar to that seen in the pED208 complex; a high-resolution structure of the protein would be useful in understanding how the Mpf complex of F-like T4SS forms and would be a new drug target for this system.

TraW is a periplasmic protein specific to the F type system that is approximately 23 kDa in size and is thought to play a role both in pilus assembly and conjugative DNA transfer [92]. TraW contains an N-terminal peptidase I signal sequence for periplasmic localization [64]. Mutations in TraW stop the pilus from extending; however, pilus tip formation occurs [14]. A previous study done with a mutated F *traW546* resulted in abolishment of the F-pilus [96]. This shows that TraW plays an essential role in the pilus extension process; however, its protein interactions are not fully understood. It is hypothesized that TraW interacts with its neighboring protein TrbC [97].

Much like TraW, TrbC is a periplasmic protein, approximately 21.5 or 23.5 kDa in size [98]. Some plasmid systems express *trbC* as a 212 aa or 23.5 kDa protein while others process the protein to remove the N-terminal signal sequence and produce a 191 aa or 21.5 kDa protein [98]. TrbC from the F-plasmid contains an N-terminal peptidase I signal sequence composed of approximately 21 aa for periplasmic localization [64]. Both TraW and TrbC are very similar in their localization and function, and in some systems, these proteins are expressed as a single polypeptide, indicating that cooperative function may occur between TraW and TrbC. Previous studies performed using conjugative mating assays showed that an interaction between the NTD of TraW and the CTD of TrbC was essential for conjugation to occur [97]. It is possible that this same interaction is required for the assembly of the pilus. When examining sequence alignments of these two proteins and comparing systems that produce two separate proteins and ones that produce one single polypeptide, it is clear that TrbC mimics the NTD of the single polypeptide while TraW mimics the CTD, further indicating an interaction between the CTD of TrbC and the NTD of TraW [97].

### 3.3. Pilus Retraction—TraH and TrbI

TraH is a cysteine-rich protein unique to F-like systems, with a size of 458 aa that is processed to 433 aa for localization, and contains a C-terminal coiled-coil domain known as a motif for oligomerization or other PPIs [14]. This motif is predicted to mediate the interaction complex between TraF, -W, -U, TrbB and -I; TraH directly interacts with TraF, TrbI and the mating pair stabilization (Mps) protein TraU [85]. TraH also contains three N-terminal hydrophobic domains of approximately 20 aa each (the first 25 aa being the cleaved signal peptide), which aligns with results showing that TraH, -F, -U and -W are bound in the OM in the context of the complex [87]. Disulfide bond formation within TraH performed by DsbA and isomerization by TrbB are important for the proper activity of TraH as shown through mutational assays. Interactions with TrbI occur at conserved sequences in the TraH N-terminus, specifically Gly193−Leu226, which also contains a Walker A motif; TraH exhibits no NTPase activity despite this characteristic motif [85,87]. As TraH has many interacting partners its role in the T4SS is difficult to ascertain; the mutation of *traH* appears to affect both pilus elongation and retraction [99].

Pilus retraction is still a major point of intrigue as it is hypothesized that it occurs in an energy independent manner [11]. Once initiated, F-pili retraction occurs at an average of 15.8 nm/s, which is less than half the mean extension rate of 39.5 nm/s [70]. TrbI is a bitopic, 128 aa IM protein that plays a role in the retraction process by interacting with the periplasmic OM-associated complex composed of TraF, -H, -U, and -W [55]. TrbI spans the IM by an N-terminal anchor from residues His17−Val40, with the remaining 88 residues forming a hydrophilic domain in the periplasmic space [85,96]. Several proteins of the T4SS that are localized to the periplasmic space express conserved cysteine residues, homologs of TrbI tend to express a single conserved cysteine residue [14]. Mutations in *trbI* were not observed to affect pilus production or DNA transfer efficiency; however, it was observed that some mutations will cause abnormally long pili, while excess TrbI has also been observed to have an effect on male-specific phage sensitivity [64]. Both single-stranded DNA and RNA phage infections are inhibited by excess TrbI [96]. Both examples support the hypothesis that TrbI functions solely in the regulation of retraction. As previously noted, TrbI can directly interact with TraH, and this interaction is considered to initiate pilus retraction [85]. If TrbI localizes in the IM and TraH assembles into the OM when in the context of the T4SS complex, the TrbI:TraH pair could be part of a second envelope-spanning structure analogous to the TraB, -K, -V core complex [87]

### 3.4. Mating Pair Formation

Two sets of genes are necessary for conjugation to proceed; MOB or mobility genes, which will be discussed in Section 5 below, and Mpf genes [100]. In general, Mpf genes are those responsible for the formation of a trans-envelope channel, which is formed to transfer the nucleoprotein complex. A mating pair is formed during pilus retraction events when donor and recipient cells are close enough such that they are no longer separated by exocellular material; thus, fusing the outer membranes [101]. Mpf between donor and recipient cells is thought to be a complex process that is not well understood for F-like systems [11]. Other well-studied T4SS, such as the P-type T pilus from *Agrobacterium tumefaciens,* have a relatively simple Mpf system, whereas F-type systems have the previously described core T4SS, as well as extra proteins involved in pilus outgrowth and retraction (TraF, -H, -U, -W, and TrbI), and additional Mps proteins, TraN and -G, which confer properties of efficient mating in liquid and on solid media.

## 4. Mating Pair Stabilization Proteins and the Dynamics of their Structural Ensemble

Mating pair stabilization (Mps) occurs after pilus retraction and Mpf events, yet these genes are not often seen outside of the F-like plasmid family; stabilization of contact between donor and recipient cells is essential for the formation of the mating pore and therefore essential for conjugation by F-like T4SS [11,102]. Other T4SS families have alternative methods for stabilizing a mating pair, such as the production of aggregation proteins, or compensation by the production and recipient-contacting of many pili in IncI plasmids [11,103,104]. The process of Mps for F-like T4SS provides properties of resistance to SDS and shear forces within a mating pair [105]. Unlike other major steps of conjugation, such as pilus assembly and retraction events, Mps is thought to be much simpler as it involves only two proteins, TraN and TraG [106]. These two proteins are predicted to span the entirety of the gram-negative bacterial cellular envelope; TraG contains an IM bound NTD and a C-terminal periplasmic domain that is hypothesized to contact TraN bound in the OM, which has loops extending into the extracellular matrix (ECM) [11]. This interaction has been deemed critical for the formation of the mating pore, as both proteins are essential for DNA transfer to occur. Despite limited knowledge of their high-resolution structures, the interaction between TraN and TraG is predicted to involve an induced fit in which the highly dynamic CTD of TraG conforms to interact with the well-structured TraN, perhaps with aid from other periplasmic T4SS components.

### 4.1. TraN

The integral OM protein TraN is arguably the primary actor for Mps as it forms essential contacts for recipient cell lipopolysaccharide (LPS) recognition prior to formation of the conjugative pore [102,106]. TraN from the F plasmid has been shown to require outer membrane protein A (OmpA) expressed in the recipient cell for Mps to occur, while TraN_R100_ can perform its action through recognition of LPS moieties alone. Binding to OmpA appears to increase mating efficiency relative to F-like plasmids that feature a TraN homolog that does not bind OmpA. TraN is hypothesized to function as a multimer, but the extent of oligomerization in the T4SS is not known as the protein is difficult to purify from the biological complex due to the low number of F pili expressed in cells and the many associations TraN forms in large complexes, as attributed to predicted intermolecular disulfide bonds [102]. Cryo-ET structures of the F-like pili pED208 did not include TraN as part of the core complex visualized (Figure 3A–C) [15]. There are 20 conserved cysteines in TraN from F-like plasmids; results suggest that these residues are involved in the behavior of this protein to act similar to an adhesin, despite its low sequence homology to adhesins with known structures [102,106,107]. Altering five of six highly conserved cysteines in the C-terminus of the protein eliminates the Mps function of TraN [102]. The number of cysteine residues in the OM segments is uncommon, which causes difficulty in the predictive modeling of TraN, as few OM proteins with solved structures contain many cysteine residues, and the presence of disulfide bonds occurring in TM segments has not been documented in biological environments [102,108].

The structure of TraN is largely hydrophobic as it is a polytopic TM protein predicted to have a β-barrel structure, although this is still highly contested based on low homology to TM β-barrel proteins, with loops extending into the ECM and in the periplasm [102,107]. Models have indicated the presence of 20 TM β sheets in the 584 aa protein (602 aa before C-terminal signal sequence processing during transport to the OM) with 10 extracellular loops and 9 loops, as well as the N-and C-termini, in the periplasm [102,109]. These loop regions have been shown to perform key interactions on both sides of the membrane. Experiments involving TraN fusions of the loop regions with c-Myc showed 3 main extracellular loops in the N-terminal half of the protein prevented interaction with OmpA, therefore these loops are responsible for the TraN-OmpA interaction [102]. Attempts to demonstrate this interaction by two-hybrid techniques were unsuccessful, as was co-immunoprecipitation from mating cells, probably due to the small number of interacting partners in a mating pair [11]. This suggests that TraN changes conformation in the context of a mating pair and interacts with OmpA, acting as a clamp to stabilize the conjugative junction. The periplasmic loops containing cysteines are predicted to perform disulfide bonding as mediated by periplasmic proteins TraF and/or TrbB, as these parts of the F pilus complex have thioredoxin-like folds [88,102,110]. They may mediate important contacts for Mps predicted to occur between the C-terminal region of TraG and TraN [102].

### 4.2. TraG and the Structural Dynamics Effectuating its Trifunctional Roles

TraG_F_ is a 102.5 kDa protein with an N-terminal TM domain bound in the IM and a periplasmic CTD from residues Ala452-Glu939 denoted TraG* [79]. It is one of the largest proteins encoded by the *tra* region of the F plasmid. Frameshift mutations in the NTD of the protein have been shown to effect the polymerization of pilus subunits, while the whole protein must be intact for Mps and entry exclusion (Eex) events to occur [79,111]. It was previously thought that the presence of a signal peptidase I cleavage site after residue Ala451 releases TraG* into the periplasm to fulfill its role in Mps, however it appears the CTD must be anchored to the IM for proper TraG* function [78,79].

The mechanism for the assembly of pilin subunits into polymers as aided by the N-terminus of TraG is not completely understood as indicated in Section 3.2.1; mutations in TraE, K, B, V, C, W, U, F, H, and the N-terminal region of TraG all result in accumulation of mature pilin in the IM [78,99]. In performing Eex, TraG is predicted to scan the neighboring recipient IM to interact with TraS, which would require the protein to interact over two OM layers. This distance can be approximated to 35–40 nm, assuming donor and recipient OMs are proximal after surface exclusion (Sfx) [112]. As this distance is likely unachievable by a single globular protein, the steps of Mps are thought occur first, and the C-terminal region of TraG is theorized to undergo a reversible conformational change and thrust into the mating pore formed after TraN interacts with OmpA of the recipient cell, potentially through interaction with TraN [79]. In this predicted model, TraG maintains an intact structure and extends TraG* through the mating pore into the periplasmic space of the recipient cell to perform its role in Eex. A TraS-TraG complex has not, however, been detected in mating cells through crosslinking or immunoprecipitation experiments, nor interaction detected using a bacterial two-hybrid system [79]. As well, interaction between TraN and TraG has not been detected using similar methods [102]. The likely cause for the lack of detectable assembly is due to the small number of interacting protein partners in a mating pair, as well as the transient nature of the PPIs.

There is a region in the aa sequence of TraG_F_ and TraG_R100_ that shows 55.7% similarity while the proteins hold an overall sequence identity of 93% [79]. Residues Thr610-Ala673 of TraG were predicted to be responsible for the trans-exclusive interaction between TraG and a cognate TraS in the Eex process, as TraG will not interact with TraS in the same IM. This region has been further specified to Thr606-Asp608 in the IncJ plasmid R391 [113]. In all cases this region is part of the periplasmic CTD, which is further evidence of the role TraG* performs in Eex. The homology between TraS_F_ and TraS_R100_ and the interaction between TraS and TraG are discussed in Section 6.2.

There is a lack of structural data for TraG from the F-like family; however, useful information is gleaned from other conjugative systems. The canonical T4SS of the IncN type plasmids is the Ti pilus from *A. tumefaciens*, largely due to the utility of this system in the genetic modification of plants mediated by the Ti plasmid [114,115]. pCRY, a similar plasmid from the same family, contains the gene product VirB8, a protein orthologous to TraG*. There is evidence indicating the evolution of the *traG* gene in the F plasmid involved the fusing of an ancestral homolog of genes *virB6* and *virB8* to produce a single gene [55]. The structure of VirB8 has been solved in numerous species; *A. tumefaciens*, *Brucella suis*, *Bartonella quintana*, *Bartonella tribocorum*, and *Rickettsia typhi* [116,117,118]. As well, TraH, a VirB8 homolog from the plasmid pIP501 (from IncN family, similar to pIPO2T) in *Enterococcus faecalis* was solved [119]. All of these structures feature a nuclear transport factor 2-like fold and show high structural homology despite low sequence homology, with backbone root mean square deviation (rmsd) values ranging between 2.6–2.8 Å in comparing all structures [119]. These VirB8 structures indicate the protein functions as a dimer; however, TraH is monomeric. A VirB8 homolog from pKM101, TraE was shown to form hexamers when isolated, and when interacting with the VirB6 homolog from this plasmid, TraD, thus, implying the diversity in oligomerization possible in these systems [120]. There are no high-resolution structures of VirB6, however structural predictions have shown there are 4–9 TM helices in VirB6 from the Ti plasmid, while the NTD of TraG is predicted to span the membrane 3–5 times [79]. These studies indicate the potential for TraG to have a C-terminus similar in structure to VirB8, and therefore may function as a monomer, a dimer, or a hexamer. As the TraG* orthologue is commonly embedded into the IM from its N-terminus in all of the described structures, it is probable that TraG* from F-like T4SS will not oligomerize into its proper complex without the membrane-bound half.

Recent studies on TraG* have shown that the region bridging the N-terminal IM bound domain and TraG* serve as a flexible linker region (N. Bragagnolo and G.F. Audette, in preparation). This was first theorized when the aa sequence for TraG was entered into the phase separation predictor (PSP) software for predicting phase separating IDRs with long range pi-pi contacts [34]. The region from residues Pro447-Gln498 was predicted to cause phase separation in the protein, with 27 of these residues surpassing the score threshold of 4.0 thus providing TraG with an overall score of 4.06. Further characterization of TraG* truncation mutants showed that this predicted phase separating region was merely highly dynamic and likely serves to allow for various conformations to be achieved by the CTD of TraG in performing its Mps and Eex functions despite being anchored to the IM (N. Bragagnolo and G.F. Audette, in preparation). Due to the multifunctional properties of TraG in the F-like T4SS, targeting this protein for rational drug design may provide new avenues for the development of antibiotics against bacterial conjugation; a high-resolution structure of the protein would greatly aid this process.

### 4.3. TraU

TraU has been generally categorized as having a role in DNA transfer as pilus synthesis is affected but not abolished by its absence, however conjugation does not proceed past the pilus retraction phase [14,59,81,121]. Phage infection is inhibited in TraU mutants indicating that its function lies in the post-adsorption phase and therefore may play a role in Mpf or Mps [79,121]. TraU is a 34 kDa protein with an N-terminal signal sequence that is processed to 33 kDa [121]. The position of the cleavage is disputed, as there exists a potential signal peptidase I cut site between Ala22-Asp23 and a signal peptidase II sequence Ser24-Ala25-Cys26. It localizes to the periplasm based on fractionation experiments; several segments of hydrophobic amino acids signify an interaction between TraU and membrane proteins in the T4SS [121,122]. There have been hypothesized interactions between TraU and TraN in the OM for Mps, and TraH in the periplasm for its effect on pilus retraction, all of which are proteins found only in F-like plasmids; no homologs are found in other T4SS [14,102,121,122]. This is further supported by the many potential redox sites in these three proteins; disulfide bond formation and cleavage may be catalyzed by TrbB and/or TraF [88]. There is currently no structural information regarding TraU, and its proposed complexes must be further studied to confirm putative functions.

## 5. The Structures of Conjugative DNA Transfer Proteins

As stated previously, bacterial conjugation is a contact dependent mechanism of transferring genetic material. Conjugative plasmids can provide their host bacterium novel virulence genes that enhance the survivability of species in their microenvironments or to inhabit new biomes. The MOB genes of conjugative plasmids code for DNA transfer proteins, which are critical components for the effective replication and assembly of the relaxosome, allowing for the rapid spread of plasmids through bacterial colonies. Presented below are the five proteins that intricately function to ensure plasmid DNA transfer occurs quickly and efficiently. Disrupting any of these proteins implicates the effectiveness of DNA transfer, and many have solved structures at high resolution, making them enticing targets for drug discovery.

### 5.1. TraY and IHF

The relaxosome accessory protein TraY is a 131 aa cytoplasmic protein and has the least structural information available of the proteins in this section. The relaxosome complex usually contains between 2 and 3 accessory proteins including, TraY, TraM, and the integration host factor (IHF), a genome encoded heterodimeric protein [3]. The origin of transfer (*oriT*) presents two binding sites for each protein, IHFA, IHFB for IHF and *sbyA* and *sbyC* for TraY [3,123]. It is presumed that TraY and IHF cooperatively stabilize the conformation of DNA that surrounds the nic site to allow TraI to bind and cleave the DNA, producing single strands [124,125,126]. Both proteins can induce a bend in bound DNA of 160° and 50–55° by IHF and TraY respectively [3]. TraY is in a class of homologous DNA-binding proteins like P22 phage repressors Arc and Mnt, which express a ribbon-helix-helix (RHH) motif in which two homodimers bind DNA [37,64]. However, TraY has been shown to bind DNA as two monomers [64,127]. The assembly of both TraY and IHF at *oriT* is required prior to TraI binding for the initiation of DNA transfer [128].

### 5.2. TraI

**Figure 5:**
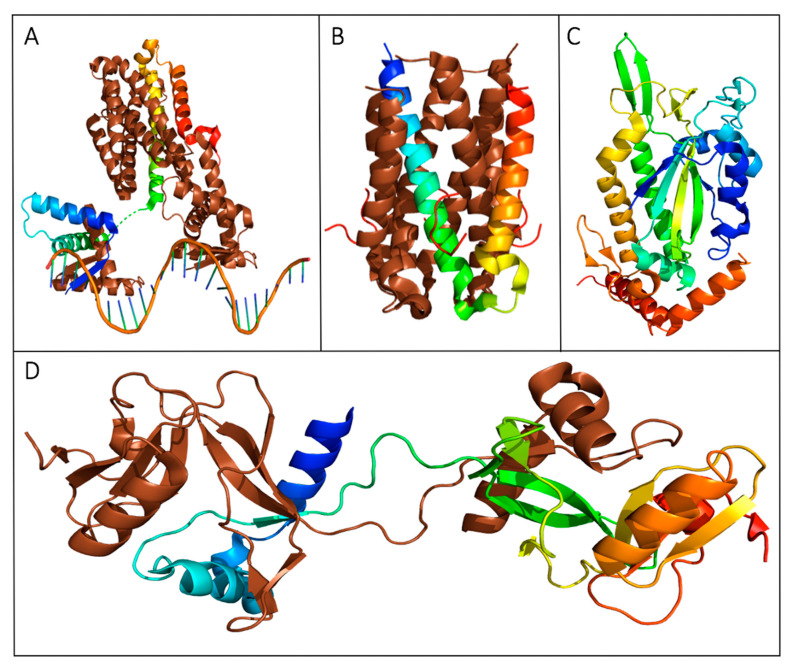
Crystal structures of transferosome proteins from the F (**A**–**C**) and pED208 (**D**) plasmids. The structures represented with oligomeric states have a monomeric unit expressed as chainbows with the remaining homo multimers represented in brown. (**A**) TraM binds three specific DNA sequences, *sbmA*, *sbmB* and *sbmC* [137]. The crystal structure of the TraM-*sbmA* interaction shows cooperative binding is facilitated by DNA distortion, allowing simultaneous binding by both tetramers while still allowing multiple contact points for TraD (PDB ID: 3ON0). (**B**) The crystal structure of the TraD–TraM interaction (PDB ID: 3D8A) [139]. TraM forms a tetrameric binding protein as represented by the monomer chainbow and the brown tetramer chains, while TraD peptides are represented in red. The C-terminal residue of TraD Phe717 binds to the hydrophobic pocket produced by TraM residues Leu85, Val106, and Ile109. (**C**) The solved crystal structure of the F plasmid TraI relaxase NTD represented in chainbows, with 10 α-helices and 13 β-strands (PDB ID: 1P4D) [134]. (**D**) The CTD of TraI forms an intertwined tetramer with two domain swapped dimers; one dimer is depicted above (PDB: 3FLD) [132]. TraI expresses a fold with a compact α-β domain at the CTD connected to two α-helices at the NTD by a proline-rich loop.

The relaxase protein, TraI is a large 1756 aa cytoplasmic protein responsible for nicking F DNA at *oriT*. This protein has proven to be bifunctional with the NTD from Met1−Pro306 presenting relaxase activity while two independent regions of Thr303−Ala844 and His830−Ser1437 corresponding to helicase folds, with the conserved motifs of DNA helicase family found in the latter fold [129,130,131]. The role played by the CTD residues Arg1476-Asp1756 of TraI is uncertain. Indirect evidence has shown that the region may interact with TraM; however, it has also been hypothesized that the domain may bind TraD to couple the relaxosome to the T4SS (Figure 5D) [132]. The N-terminal relaxase domain contains two pairs of adjacent tyrosines, which are thought to catalyze the cleavage reaction performed by TraI, however Tyr16 can act as a lone catalytic tyrosine to ensure DNA transfer occurs and has been shown to be essential to conjugation [1,130,133]. The high-resolution structure of the relaxase domain of TraI has been resolved by X-ray crystallography (Figure 5C) [134]. The relaxase domain of TraI binds to ssDNA in a bent conformation in which mainly α-helical domain borders the β-sheet [131,135]. In order for cleavage to occur a divalent metal ion like Mn^2+^, Zn^2+^, Ni^2+^, Cu^2+^, Ca^2+^, etc., must be present [136]. TraI binds specifically to the site and strand, catalyzing a transesterification reaction at *oriT,* and ‘nicking’ the DNA at the 5′ end. TraI helicase then unwinds the DNA in 5′ → 3′ direction to allow for ssDNA to be transferred, as mediated by the TraM signal protein [37,129]. The structure of TraI is conserved within the F-like plasmid family and is currently considered as a useful target for novel antibiotics due to its ubiquitous nature in the conjugation process [1].

### 5.3. TraM

The signal protein, TraM is a 127 aa cytoplasmic protein thought to provide the signal required to unwind DNA by the *oriT* nicking machinery [64]. TraM is capable of binding to three genes within the *oriT*; *sbmA*, *sbmB,* and *sbmC* with the interaction between TraM and *sbmC* being essential to its interaction with the relaxosome allowing for the efficient mobilization of plasmids [123,124]. TraM has highest affinity for the *sbmA* gene; the structure of this interaction has been determined to high-resolution (Figure 5A) [137]. Sites *sbmA* and *sbmB* overlap the promoter for the *traM* gene and *sbmC* is found within the *oriT* sequence [124]. The F TraM protein is tetrameric and composed of approximately 79% α-helix, with the remainder exhibiting β-strands [137]. The CTD has been observed to tetramerize and is the region responsible for interactions with TraD, while the NTD homodimerizes to form a ribbon-helix-helix (RHH) DNA-binding motif. TraM proteins have been found to bind specific sites on the top strand of F DNA following nicking at the *oriT* [138]. A single TraM tetramer binds DNA and induces unwinding to allow recognition by the RHH domain; the DNA is then kinked by the protein leaving it in a high-energy state, at which point a second TraM tetramer binds to stabilize the complex [137]. Pairs of TraM tetramers cooperatively bind DNA without further homo-multimerizing, allowing their CTDs to interact with TraD.

### 5.4. TraD

The essential coupling protein TraD is a 717 aa IM protein that plays a key role in the transfer of ssDNA during conjugation; it is a component of the transferosome. The most notable feature of the coupling protein is the flexible C-terminal tail that extends into the cytoplasm, considered responsible for relaxosome recognition in the DNA transfer process. The CTD of TraD, more specifically Ala680-Phe717, is highly conserved in other members of the F-like plasmid family [139,140]. The typical structure of a coupling protein is composed of an N-terminal TM domain and a large cytoplasmic region composed of an α-helical bundle and a nucleotide binding domain (NBD); these traits have been observed in TraD [55]. Three features common to all F-like TraD proteins include (1) a glycine residue within the tail which is essential in the formation of a crucial β-turn, (2) an aromatic residue present at the end of the chain, (3) a high percentage of acidic residues [139]. Mutations of the *traD* gene cause plasmid transfer deficiency [37,64]. Although the structure of TraD_F_ has not been resolved, the soluble fragment of the TraD homolog TrwB of R388 has been solved by X-ray crystallography; thereby little structural information is present for the TM domain [141]. The hexameric ring structure of TrwB is divided into two domains, one composed of an all α-helical domain (AAD) and another closer to the membrane composed of an α/β twisted open-sheet domain [59,141]. Although purified TraD has been shown to bind DNA in a nonspecific manner, the relaxosome-transferosome interaction observed between TraD and TraM provides a degree of specificity [55].

The temporary binding between TraD and TraM has been observed by the recruitment of TraM by the flexible C-terminal tail displayed by TraD (Figure 5B) [138,139]. As previously stated, the C-terminal tetramerization domain of TraM interacts with residues Asp710-Phe717 of the CTD of TraD [37,137]. This interaction involves hydrophobic and hydrophilic contacts, Phe717 present at the end of the CTD of TraD is observed to interact with the hydrophobic pocket produced by TraM (Leu85, Val106, and Ile109), and the carboxyl group anchors the TraD in place through interaction with Arg110 and Lys76 [139]. This interaction is highly conserved in other F-like plasmids and is essential to successful DNA transfer as it is thought to allow for the coupling of the relaxosome to the transferosome [123,138,140].

## 6. Exclusion Proteins and their Low Structural Dynamicity

Alongside Mps, yet prior to the formation of the mating pore, exclusion steps occur to prevent redundant plasmid transfer to a recipient that already holds the same element. Sfx proceeds first and is mediated by the OM lipoprotein TraT expressed on the cell surface; if TraT is present in the recipient cell’s membrane, then the formation of mating aggregates is inhibited and conjugation will be prevented [105,142]. If Sfx fails to prevent mating aggregate formation and Mps steps proceed, then Eex is relied upon to avert redundant donor-donor transfer. The IM protein TraS and the periplasmic C-terminal half of TraG are interacting partners in the Eex event; if the potential recipient has the same plasmid, TraG in the donor cell is thought to scan the IM of the recipient cell and interact with the cognate TraS to arrest conjugation [79]. The cells will remain in an aggregate if the colony is highly dense (however they are separable by SDS and shear forces unlike Mps-stabilized aggregates), and if the environment is diffuse the cells will separate and drift apart [105]. If the recipient cell does not have a cognate TraS in its IM, the C-terminus of TraG is predicted to extend to contact TraN in the OM and further the formation of the mating junction [106].

Exclusion genes have proven to be important for the stability of a conjugative plasmid; F mutants with both *traS* and *traT* mutations are unstable and have not been isolated [105,143]. This is supported by F plasmid-mediated superinfection immunity, which was determined to be mediated by TraT and TraS [144]. Superinfection refers to the process of lethal zygosis, a phenomenon that was seen when an excess of high frequency of recombination (Hfr) F donors were placed in contact with F¯ recipients; many of the recipient cells died [145]. The presence of TraS however was deemed to be more important for colony survival than TraT as Eex by TraS and TraG also provide a higher exclusion index (EI) than Sfx by TraT [142,144]. EI is calculated as the frequency of transfer of a plasmid given to a plasmid-free recipient divided by the frequency of transfer to a recipient carrying the same plasmid. For the F-plasmid in *E. coli,* mating was observed at 100–300 times higher frequency when the plasmid was not present in the recipient in comparison to donor-donor exchange; thus, the EI was 100–300 [105]. In comparing EI of donor-recipient exchanges for the F plasmid, *traT* point mutants resulted in a smaller reduction in mating than the *traS* point mutants [143]. An increased exclusion activity was seen in the Eex system, which was attributed have an EI 500–1000-fold greater than that associated to the Sfx system, indicating a higher reliance on the Eex system for preventing erroneous donor-donor transfers [11,79,142]. The exclusion process performed by these systems is also seen to be gene-dose dependent; when *traS* and *traT* were cloned into a multicopy plasmid, the EI increased to 10,000 [105].

### 6.1. TraT

The only protein known to participate in Sfx, TraT is an OM bound protein that is well structured and displays high homology in F-like plasmids [11,105]. TraT is processed prior to forming its active conformation in the OM; proTraT contains a 21 aa hydrophobic signal sequence that is modified through esterification by a diacylglycerol in Cys22, then the signal sequence is cleaved off the N-terminus to form a 233 aa protein [146,147,148]. The addition of a third acyl group at the amide forms the mature TraT lipoprotein at 23,709 Da. TraT is considered to be a major OM protein as it is highly expressed in F+ cells with more than 20,000 copies per cell; the protein forms multimeric aggregates to perform its function [105,147]. ProTraT has been shown to translocate into the OM; however, it does not oligomerize resulting in a lack of function [148]. As TraT is a lipoprotein its strong anchoring into the membrane is formed by intermolecular lipid-lipid interactions and lipid-protein interactions [142,146]. This provides high structural stability to the protein as TraT can withstand denaturation by high temperatures, solubilization by detergents and proteolytic cleavage by proteases [148].

In comparing TraT_F_ to TraT, from all known IncF plasmids, the lowest homology between the aa sequence of the mature protein is 80% (in comparing to TraT_pED208_), and the highest homology is held with TraT_R100_ which only differs by 1 aa [142,149]. Although residue 120 is a glycine in TraT_F_ and alanine in TraT_R100_, Sfx limits conjugation between cultures containing the two different plasmids 3-fold less than if mated with bacteria containing the same plasmid. Structural predictions indicate that the single residue change causes an extra β-turn that radically alters the local structure of the domain. The 5 aa region from residues Arg116-Gly120 has been experimentally shown to be important for Sfx specificity and is known to be a hydrophilic exposed region in the ECM [150,151]. Therefore, these residues are considered responsible for the interaction with TraT and its unknown binding partner. TraT was initially proposed to define the specificity of the system through the formation of a 5 membered ring, with the extracellular motif acting as a recognition sequence for an incoming pilus tip and blocks its interaction with OmpA [152]. The sequence variability of pilins cannot provide the required specificity based on experimental results, thus a new mechanism of Sfx was proposed where TraT disrupts the Mps interactions of TraN with OmpA [102,106,153]. While evidence of interaction between TraT and OmpA exists, this mechanism has not been confirmed experimentally [106,142,152]. An interaction with TraN is plausible as TraT is involved in disaggregating mating pairs after DNA transfer in a mechanism independent from its function in the disruption of donor-donor conjugation [11,154].

No high-resolution structural information is available for TraT from F-like plasmids, however TraT_F_ was predicted to have 55% α-helical content [147]. It is also believed to differ radically in molecular organization in the OM compared to other OM proteins based on its unusually long stretches of uncharged residues (Val99-Ser110 and Tyr118-Asn135) that likely contribute to TM helices. Analysis of TraT_F_ sequence using Phyre2 indicated no homologs have been deposited in the PDB, implying the novelty of the TraT sequence [20]. In analyzing the available structural information, an interesting pattern is seen in which the high structural integrity of the protein results in high sensitivity to slight mutations, which are likely to drastically alter the protein’s structure. TraT may be a useful drug candidate as slight structural changes that could be induced by a small molecule would likely result in loss of Sfx function.

### 6.2. TraS

TraS is a small, highly hydrophobic IM protein which functions solely in Eex through interaction with TraG, no other TraS interactions have been proposed to occur [79,81]. The 149 aa protein has not been studied thoroughly as it is difficult to express and purify; the only hydrophilic domain is from residues Glu10-Arg24 [79,155]. Unlike TraT, TraS does not appear to have a signal sequence and evidently is not a lipoprotein [156]. In further comparing the protein to TraT, sequence conservation between different F-like plasmids is much lower. TraS_F_ and TraS_R100_ have the highest sequence identity at 17%, and where TraT_F_ had its lowest protein sequence identity to TraTpED208, TraS_pED208_ has no sequence homology to TraS_F_ [79]. This diversification in protein sequence may serve as the reason for the 500–1000-fold higher effect of blocking mating by Eex in comparison to the Sfx system [11,79]. As mentioned previously, Mps has been suggested to involve interaction of TraG in the donor cell with the IM of the recipient, thereby fusing the cells together [11]. This could be interrupted by TraS and therefore be the mechanism by which Eex prevents an essential step in the formation of the conjugative pore for F-like T4SS.

As few structural studies on TraS from F-like plasmids have been performed, structural predictions can only be made based on other forms of data. As mentioned, TraS_F_ and TraS_R100_ hold low sequence identity and participate in entry exclusion when conjugative mating assays are performed with chimeras of non-cognate TraG [79]. As chimeras of TraS have not been expressed successfully due to their instability, likely due to membrane insertion issues, no predictions have been made about the region that exhibits TraG-binding specificity. The number of TM domains in TraS_F_ and TraS_R100_ are predicted to be similar (3 or 4); however, their locations in the position of the sequence are significantly different. Another study hypothesized that Eex specificity lies in the C-terminal half of TraS from the R391 plasmid, but this has not been confirmed in TraS from F-like plasmids [113].

Entering the TraS_F_ sequence into prediction software such as Phyre2 results in similar predictions: four TM helices are present, and the hydrophilic helix in the N-terminal is recognized as it was the only region modeled, but with poor certainty (38% confidence) [20]. The lack of homologs for the TM portion of TraS is indicative of the absence of structural data for small TM proteins in the PDB. The protein is predicted to be highly structured and all α-helical (90% of total sequence); thus, displaying how a highly dynamic protein TraG interacts with a well-structured protein TraS to perform its function, likely indicating an induced conformation upon the interaction of TraG with TraS in the IM of the recipient cell. The TraG-TraS interaction is a target for manipulation by novel drugs, as recent studies have shown the most notable genetic difference linking plasmid exclusion to the slow development of plasmid-mediated antibiotic resistance was the presence of a functional *traS*-family gene for Eex events [157].

## 7. Conclusions

In analyzing the known structural data of F-like plasmid proteins from the *tra* operon, some trends have emerged. Cytoplasmic proteins have been well-studied and have high-resolution structures, while little structural insight is known for proteins that are part of the T4SS complex; this is expected based on the difficulty to purify and crystallize proteins, which are part of large complexes and contain hydrophobic regions. The solved cytoplasmic proteins have either a T4SS transcription regulatory role or a role in conjugative DNA transfer, and are confirmed to have a moderate degree of disorder in their structure; however, this degree of flexibility is common in bacterial DNA-binding proteins [158,159].

The periplasmic proteins involved in complexes have a diminutive level of structural data and only distant homologs with solved structures in the PDB, despite a large body of biochemical information. The identity of the highest confidence models as determined through Phyre2 are below 20% for many of these proteins, and are all models of partial sequences less than half the size of the protein (Table 1; Table S1) [20]. As many of these proteins are vital for conjugation, elucidating their structures would greatly aid in determining their precise role and interacting partners, and provide novel structures to shared databases. Of the IM proteins involved in pilus assembly and extension, TraE, -P, -L, and the NTD of TraG have unconfirmed function. The only other Tra protein discussed with unconfirmed function is TraU; all described proteins would have novel folds based on their low homology to solved structures.

Structure-based drug discovery is considered to be the most efficient method proposed for the development of novel conjugation inhibitors [2]. As mentioned Section 5.2, the relaxase TraI was determined to be a druggable protein; however, small molecule ligand inhibitors belonging to the bisphosphonate family of compounds were determined to function as non-specific chelating agents [1,160]. Specific single-chain F_V_ antibody inhibitors have been produced to target TraI homologs, however their expression requires a recipient transgenic population and each inhibitor would only function on the cognate relaxase [161]. Development of small molecule ligand inhibitors that successfully inhibit bacterial relaxases from plasmid families or subfamilies would be optimal; if a full structure of TraI were solved, structural-based drug discovery would be facilitated for the IncF plasmid family. The TraG homolog VirB8 has a proposed druggable site at a PPI interface (Section 4.2), however small molecule ligands proven to be optimal for disrupting conjugation in Ti plasmid donors show poor inhibitory action in the related TraE from pKM101 [2,117,162]. Exploiting the Fin-TraJ expression system through the use of activators or inhibitors would be highly effective (discussed in Section 2), however drugs targeting these biomolecules are likely to suffer from pronounced plasmid specificity [163]. The ATPase TrwD from the R388 plasmid has been inhibited through the administration of unsaturated fatty acids (UFAs) such as linoleic acid, and 2-hexadecanoic acid; docking simulations have provided context for further optimization as the UFAs bind non-competitively [164]. TrwD is part of a superfamily of ATPases that include the Type II secretion, Type IV pilus and flagellar biogenesis machineries all of which have putative binding sites for similar UFAs [2]. It is possible that UFAs may be found that can target TraC from the F-like T4SS (Section 3.2.3); a high-resolution structure of TraC would greatly aid in future in silico drug discovery processes. An increasingly promising drug target is the pilus itself; as mentioned previously, structural data exists for the polymerized pilus, and many functional studies have been performed on pili from a vast number of plasmids. Bacteriophages have high affinity for the bacterial pilus, and the M13 phage was shown to bind F-type pili using the coat protein g3p [165,166]. This interaction allowed for the creation of a potential protein antibiotic, as addition of the soluble N-terminal domain of g3p to F-plasmid containing bacteria resulted in inhibition of conjugation [167]. Many drugs have been found to prevent conjugation as discovered through high-throughput screening assays, such as peptidomimetic compounds that have been shown to disrupt conjugative DNA transfer; however, their sites of inhibition are unknown [2,168]. Structural data of T4SS components would facilitate the development of docking simulations, enabling the discovery of the proposed binding sites such that the discussed small molecule ligands can be further optimized.

In examining the dynamics of the proteins that compose the F-like T4SS, structural data and Phyre2 predictions show that many proteins have disordered regions. The highest percentage of disordered sequences within a functional grouping were found in the core T4SS proteins TraB, -K, and -V, with 44, 34, and 61% respectively. Although these proteins have homologs with structures solved to low resolution by cryo-ET, a lack of homologs with high-resolution models may be the reason for the predicted level of disorder by Phyre2 rather than these proteins being intrinsically disordered [15,169]. In general, the presence of highly dynamic regions in these proteins may be the reason for many having multi-functional abilities, and for their ability to form complexes with each other in a specific manner [170,171]. Targeting these flexible regions with small molecule drugs may be an efficacious option to develop novel antibiotics, which disrupt the conjugative T4SS. More structural information is needed to achieve efficient in silico drug design regarding proteins in the T4SS complex; those biomolecules that regulate its expression and form the transferosome complex are currently effective drug targets.

## Figures and Tables

**Table 1:** Predicted Structural Dynamics of T4SS Transfer Proteins of the F Plasmid *.

					Highest Confidence Model		
Function	Protein	Localization	Size (aa)	% Disordered	Sequences	% Identity	PDB
Transcription Regulation	FinO	Cytoplasm	186	42%	33–184	100%	1DVO
	TraJ	Cytoplasm	229	21%	15–128	100%	4KQD
	TraR	Cytoplasm	73	19%	4–73	100%	5W1S
ProPilin Maturation	TraA	IM, ECM	70	20%	21–41	29%	2L8S
	TraQ	IM	94	22%	32–64	46%	3MP7
	TraX	IM	248	13%	28–37	24%	2KP6
T4SS Core Proteins	TraB	IM, Periplasm	475	44%	203–412	14%	6GYB
	TraK	OM, Periplasm	242	34%	26–107	8%	6GYB
	TraV	IM	171	61%	25–36	42%	5V8K
Pilus Assembly/Extension	TraP	IM	196	24%	57–118	18%	2V4J
	TraE	IM	188	11%	93–182	16%	5KPE
	TraL	IM	91	13%	18–47	30%	3GEB
	TraC	Cytoplasm, IM	875	15%	448–819	18%	4AG5
	TraW	Periplasm	210	16%	100–171	20%	2FU3
	TrbC	Periplasm	212	29%	103–190	13%	1HYU
	TraF	Periplasm	247	17%	146–256	19%	6GC1
	TrbB	Periplasm	181	31%	33–175	19%	2HYX
Pilus Retraction	TraH	OM, Periplasm	458	25%	90–144	24%	6SZ9
	TrbI	IM, Periplasm	128	23%	47–120	81%	1U2M
Mating Pair Stabilization	TraN	OM, Periplasm	602	25%	127–408	98%	4XBM
	TraG	IM, Periplasm	938	43%	774–930	93%	3CWG
	TraU	Periplasm	330	26%	31–39	26%	3TOW
Relaxase	TraI	Cytoplasm	1756	20%	1–1473	98%	5N8O
Relaxosome Accessory	TraY	Cytoplasm	131	25%	1–51	25%	1U9P
	TraM	Cytoplasm	127	33%	2–121	39%	3ON0
	TraD	Cytoplasm, IM	717	29%	145–574	30%	1E9R
Surface Exclusion	TraT	OM, ECM	244	34%	52–98	15%	3BF2
Entry Exclusion	TraS	IM	173	8%	22–35	38%	2M4V

* Based on structural predictions in Phyre2 [20]. Shown is the percentage of disordered content based on secondary structure predictions and highest confidence models as determined through sequence homology of structures deposited in the PDB. Refer to Table S1 for further details regarding disordered sequences.

## Supplementary Materials

Supplementary materials can be found at https://www.mdpi.com/2227-9059/8/9/362/s1.
