# Supplementary material for: Protein Dynamics in F-like Bacterial Conjugation
Source: Biomedicines. 2020 Sep 19;8(9):362. doi: 10.3390/biomedicines8090362 (PMC7555446; doi:10.3390/biomedicines8090362)
Supplement: Supplementary file 1 [file biomedicines-08-00362-s001.pdf]

Supplementary Information

Protein Dynamics in F-like Bacterial Conjugation

Nicholas Bragagnolo, Christina Rodriguez, Naveed Samari-Kermani, Alice Fours, Mahboubeh Korouzhdehi, Rachel Lysenko and Gerald F. Audette[†]

Department of Chemistry & Centre for Research on Biomolecular Interactions

York University, 4700 Keele St., Toronto, ON M3J 1P3 Canada

Supplementary Table 1: Details Regarding Predicted Disordered Regions of Transfer Proteins from the F Plasmid*

Protein	Size (aa)	Disorder (%)	GenBank	UniProtKB	AA Sequence
FinO	186	42%	M38047	P22707	MTEQKRPVLTLKRKTEGETLVRSRKTIINVTTPPKWKVKKQKLAEKAAREAELAAKKAQARQALS<br>IYLNLPTLDDAVNTLKPWWPGLFDGDTPRLLACGIRDVLLEDVAQRNIPLSHKKLRRALKAITRSES<br>YLCAMKAGACRYDTEGYVTEHISQEEEAYAAERLDKIRRQNRIKAELQAVLDEK
TraJ	229	21%	BAA97942	P06626	MYPMDRIQQKHARQIDLLENLTAVIQDYPNPACIRDETGKFIFCNTLFHESFLTQDQSAEKWLLSQR<br>DFCELISVTEMEAYRNEHTHLNLVEDVFIQNRFWTISVQSFLNGHRNIILWQFYDAAHVRHKDSYN<br>QKTIVSDDIRNIIRRMSDDSSVSSYVNDVFYLYSTGISHNAIARILNISISTSKKHASLICDYFSVSNKD<br>ELIILLYNKKFIYYLYEKAMCIINTR
TraA	70	20%	BAA97944	P04737	AGSSGQDLMASGNTTVKATFGKDSSVVKWVVLAEVLVGAVMYMMTKNVKFLAGFAIISVFIAVG<br>MAVVGL
TraR	73	19%	BAA97953	P41065	MSDEADEAYSVTEQLTMTGINRIRQKINAHGIPVYLCEACGNPIPEARRKIFPGVTLCVECQAYQER<br>QRKHYA
TraQ	94	22%	BAA97964	P18033	MISKRRFSLPRLDITGMWVFSLGVWFHIVARLVYSKPWMAFFLAELIAAILVLFGAYQVLDAWIAR<br>VSREEREALEARQQAMMEGQQEGGHVSH
TraX	248	13%	BAA97975	P22709	MTTDNTNTTRNDSLVARTDTWLQSLLVWSPGQRDIIKTVALVLMVLDHANRILHLDQSWMFLAG<br>RGAFPLFALVWGLNLSRHAHIRQLAINRLWGWAVFSQFWYYFAGFPWYEGNILFAFAVAAQVLT<br>WCETRSGWRTAAAILMMALWGPLSGTSYGIAGLLMLAVSHRLYRAEDRMERLVLVACLLAVIPA<br>LNLATSDAAAVAGLVMTVLTVGLVSCAGKLLSRFWPGDFFPTFYACHLAVLGVLAL
TraB	475	44%	BAA97948	P41067	MASINTIVKRKQYLWLGIVVVGTASAIGGALYLSDVDMSGNGETVAEQEPVPDMTGVVDTTFDDK<br>VRQHATTEMQVTAAQMQKQYEEIRRELDVLNKQRGDDQRRIEKLGQDNAALAEQVKALGANPV<br>TATGEPVPQMPASPPGPEGEPQPGNTPVSFPPQGSVAVPPPTAFYPGNGVTPPPQVTYQSVPVPNRIQ<br>RKVFTRNEGKQGPSLPYIPSGSFAKAMLIEGADANASVTGNESTVPMQLRITGLVEMPNSKTYDAT<br>GCFVGLEAWGDVSSERAIVRTRNISCLKDGKTIDMPIKGHVSFRGKNGIKGEVVMRNGKILGWAW<br>GAGFVDGIGQGMERASQPAVGLGATAAYGAGDVLKMGIGGGASKAAQTLSDYYIKRAEQYHPVI<br>PIGAGNEVTVVFQDGFQLKTVEEMALERTQSRAEEDNPESPVPVPPSAESHLNGFNTDQMLKQLGN<br>LNPQQFMSGSQGGGNDGK
TraK	242	34%	BAA97947	P41066	MRKNNTAIIFGSLFFSCSVMAANGTLAPTVVPMVNGGQASIAISNTSPNLFTVPGDRIIAVNSLDGAL<br>TNNEQTASGGVVVATVNKKPFTFILETERGLNLSIQAVPREGAGRTIQLVSDLRGTGEEAGAWETS<br>TPYESLLVTISQAVRGGKLPAGWYQVPVTKETLQAPAGLSSVADAVWTGNHLKMVRFAVENKTL<br>SALNIRESDFWQPGTRAVMFSQPASQLLAGARMDVYVIRDGEGN
TraV	171	61%	BAA97952	P41069	MKQTSFFIPLLGTLLLYGCAGTSTEFECNATTSDTCMTMEQANEKAKKLERSSEAKPVAASLPRLA<br>EGNFRTMPVQTVTATTPSGSRPAVTAHPEQKLLAPRPLFTAAREVKTVVPVSSVTPVTPPRPLRTGE<br>QTAALWIAPYIDNQDVYHQPSSVFFVIKPSAWGKPRIN

TraF	247	17%	BAA97961	P14497	MNKALLPLLLCCFIFPASGKDAGWQWYNEKINPKEKENKPVPAAPRQEPDIMQKLAALQTATKRA<br>LYEAILYPGVDNFVKYFRLQNYWAQQAGLFTMSARKAMLAHPELDYNLQYSHYNGTVRNQLAA<br>DQAQQRQAIAKLAEHYGIMFFYRGQDPIDGQLAQVINGFRDTYGLSVIPVSVDGVINPLLPDSRTDQ<br>GQAQRLGVKYFPAMMLVDPKQGSVRPLSYGFISQDDLAKQFLNVSEDFKPNF
TrbB	181	31%	BAA97965	P18035	MSLTKSLLFTLLLSAAAVQASTRDEIERLWNPQGMATQPAQPAAGTSARTAKPAPRWFRLSNGRQ<br>VNLADWKVVLFMQGHCPYCHQFDPVLKQLAQQYGFSVFSYTLDGQGDTAFPEALPVPPDVMQTF<br>FPNIPVATPTTFLVNVNTLEALPLLQGATDAAGFMARVDTVLQMYGGKKGAK
TraC	875	15%	BAA97954	P18004	MNNPLEAVTQAVNSLVTALKLPDESAKANEVLGEMSFPQFSRLLPYRDYNQESGLFMNDTTMGF<br>MLEAIPINGANESIVEALDHMLRTKLPRGIPLCIHLMSSQLVGDRIEYGLREFSWSGEQAERFNAITR<br>AYYMKAAATQFPLPEGMNLPLTLRHYRVFISYCSPSKKKSRADILEMENLVKIIRASLQGASITTQT<br>VDAQAFIDIVGEMINHNPDSLYPKRRQLDPYSDLNYQCVEDSFDLKVRADYLTLGLRENGRNSTAR<br>ILNFHLARNPEIAFLWNMADNYSNLLNPELSISCPFILTLTLVVEDQVKTHSEANLKYMDLEKKSKT<br>SYAKWFPSVEKEAKEWGELRQRLGSGQSSVVSYFLNITAFCKDNNETALEVEQDILNSFRKNGFELI<br>SPRFNHMRNFLTCLPFMAGKGLFKQLKEAGVVQRAESFNVANLMPLVADNPLTPAGLLAPTYRNQ<br>LAFIDIFFRGMNNTNYNMAVCGTSGAGKTGLIQPLIRSVLDSGGFAVVFDMGDGYKSLCENMGGV<br>YLDGETLRFNPFANITDIDQSAERVRDQLSVMASPNGNLDEVHEGLLLQAVRASWLAKENRARID<br>DVVDFLKNASDSEQYAESPTIRSRLDEMIVLLDQYTANGTYGQYFNSDEPSLRDDAKMVVLELGG<br>LEDRPSLLVAVMFSLIIYIENRMYRTPRNLKKLNVIDEGWRLLDFKNHKVGEFIEKGYRTARRHTG<br>AYITITQNIVDFDSDKASSAARAAWGNSSYKIILKQSAKEFAKYNQLYPDQFLPLQRDMIGKFGAA<br>KDQWFSSFLLQVENHSSWHRLFVDPLSRAMYSSDGPDFEFVQQKRKEGLSIHEAVWQLAWKKSGP<br>EMASLEAWLEEHEKYRSVA
TraW	210	16%	BAA97956<br>(cont.)	P18472	MRCRGLIALLIWGQSVAAADLGTWGDLWPVKEPDMLTVIMQRLTALEQSGEMGRKMDAFKERVI<br>RNSLRPPAVPGIGRTEKYGSRLFDPSVRLAADIRDNEGRVFARQGEVMNPLQYVPFNQTLYFINGD<br>DPAQVAWMKRQTPPTLESKIILVQGSIPEMQKSLDSRVYFDQNGVLCQRLGIDQVPARVSAVPGDR<br>FLKVEFIPAEEGRK
TrbC	212	29%	BAA97958	P18473	MKLSMKSLAALLMMLNGAVMASENVNTPENRQFLKQQENLSRQLREKPDHQLKAWAEKQVLEN<br>PLQRSDNHFLDELVRKQQASQDGKPRQGALYFVSFSIPEEGLKRMLGETRHFGIPATLRGMVNNDL<br>KTTAEAVLSLVKDGATDGVQIDPTLFSQYGIRTVPALVVFCSQGYDIIRGNLRVGQALEKVAATGD<br>CRQVAHDLLAGKGDSGK
TraP	196	24%	BAA97949	P41068	MANNMSSRQACHAARYVVARVLRGLFWCLKYTVILPLATMALMALFVLWKDNTTPGKLLVKEI<br>NFVRQTAPAGQFPVSECWFSSSDSSGRSEIQGICHYRAADAADYVRETDRSLMQLVTALWATLAL<br>MYVSLAAITGKYPVRPGKMKCIRVVTADEHLKEVYTEDASLPGKIRKCPVYLPDDRTNRNNGDKN<br>EHA
TraE	188	11%	BAA97946	P08322	MEHGARLSTSRVMAIAFIFMSVLIVLSLSVNVIQGVNNYRLQNEQRTAVTPMAFNAPFAVSQNSAD<br>ASYLQQMALSFIALRLNVSSETVDASHQALLQYIRPGAQNQMKVILAEEAKRIKNDNVNSAFFQTS<br>VRVWPQYGRVEIRGVLKTWIGDSKPFTDIKHYILILKRENGVTWLDNFGETDDEKK
TraL	91	13%	BAA97945	P08321	MSGDENKLKKYRFPETLTNQSRWFGLPLDELIPAAICIGWGITTSKYLFGIGAAVLVYFGIKKLKKG<br>RGSSWLRDLIYWYMPTALLRGIFS

TraH	458	25%	BAA97968	P15069	MMPRIKPLLVLCAALLTVTPAASADVNSDMNQFFNKLGFASNTTQPGVWQGQAAGYAYGGSLYA<br>RTQVKNVQLISMTLPDINAGCGGIDAYLGSFSFINGEQLQRFVKQIMSNAAGYFFDLALQTTVPEIK<br>TAKDFLQKMASDINSMNLSSCQAAQGIIGGLFPRTQVSQQKVCQDIAGESNIFADWAASRQGCTVG<br>GKSDSVRDKASDKDKERVTKNINIMWNALSKNRMFDGNKELKEFVMTLTGSLVFGPNGEITPLSA<br>RTTDRSIIRAMMEGGTAKISHCNDSDKCLKVVADTPVTISRDNALKSQITKLLASIQNKAVSDTPLD<br>DKEKGFISSTTIPVFKYLVDPQMLGVSNSMIYQLTDYIGYDILLQYIQELIQQARAMVATGNYDEAV<br>IGHINDNMNDATRQIAAFQSQVQVQQDALLVVDRQMSYMRQQLSARMLSRYQNNYHFGGSTL
TrbI	128	23%	BAA97955	P18006	MSSTQKPADVTAERRSHWWWTVPGCLAMVLLNAAVSYGIVRLNAPVTVAFNMKQTVDAFFDSA<br>SQKQLSEAQSKALSARFNTALEASLQAWQQKHHAVILVSPAVVQGAPDITREIQQDIARRMRAEP
TraN	602	25%	BAA97959	P24082	MKRILPLILALVAGMAQADSNSDYRAGSDFAHQIKGQGSSSIQGFKPQESIPGYNANPDETKYYGG<br>VTAGGDGGLKNDGTTEWATGETGKTITESFMNKPKDILSPDAPFIQTGRDVVNRADSIVGNTGQQC<br>SAQEISRSEYTNYTCERDLQVEQYCTRTARMELQGSTTWETRTLEYEMSQLPAREVNGQYVVSITS<br>PVTGEIVDAHYSWSRTYLQKSVPMTITVLGTPLSWNAKYSADASFTPVQKTLTAGVAFTSSHPVRV<br>GNTKFKRHTAMKLRLVVRVKKASYTPYVVWSESCPFSKELGKLTKTECTEAGGNRTLVKDGQSY<br>SMYQSCWAYRDTYVTQSADKGTCQTYTDNPACTLVSHQCAFYSEEGACLHEYATYSCESKTSGK<br>VMVCGGDVFCLDGECDKAQSGKSNDFAEAVSQLAALAAAGKDVAALNGVDVRAFTGQAKFCKK<br>AAAGYSNCCKDSGWGQDIGLAKCSSDEKALAKAKSNKLTVSVGEFCSKKVLGVCLEKKRSYCQF<br>DSKLAQIVQQQGRNGQLRISFGSAKHPDCRGITVDELQKIQFNRLDFTNFYEDLMNNQKIPDSGVLT<br>QKVKEQIADQLKQAGQ
TraG	938	43%	BAA97969	P33790	MNEVYYVIAGGEWLRNNLNAIAAFMGTWTWDSIEKIALTLSVLAVAVMWVQRHNVMDLLGWVA<br>VFVLISLLVNVRTSVQIIDNSDLVKVHRVDNVPVGLAMPLSLTTRIGHAMVASYEMIFTQPDSVTYS<br>KTGMLFGANLIVKSTDFLSRNPEIINLFQDYVQNCVLGDIYLNHKYTLEDLMASADPYTLIFSRPSPL<br>RGVYDNNNNFITCKDASVTLKDRLNLDTKTGGKTWHYYVQQIFGGRPDPDLLFRQLVSDSYSYFY<br>GSSQSASQIMRQNVTMNALKEGITSNAARNGDTASLVSLATTSSMEKQRLAHVSIGHVTMRNLPM<br>VQTILTGIAIGIFPLLILAAVFNKLTLSVLKGYVFALMWLQTWPLLYAILNSAMTFYAKQNGAPVVL<br>SELSQIQLKYSNLASTAGYLSAMIPPLSWMMVKGLGAGFSSVYSHFASSSISPTASAAGSVVDGNYS<br>YGNMQTENVNGFSWSTNSTTSFGQMMYQTGSGATATQTRDGNMVMDASGAMSRLPVGINATRQ<br>IAAAQQEMAREASNRAESALHGFSSSIASAWNTLSQFGSNRGSSDSVTGGADSTMSAQDSMMASR<br>MRSAVESYAKAHNISNEQATRELASRSTNASLGLYGDAYAKGHLGISVLGNGGGVGLQAGAKASI<br>DGSDLDSHEASSGSRASHDARHDIDARATQDFKEASDYFTSRKVSESGSHTDNNADSRVDQLSAAL<br>NSAKQSYDQYTTNMTRSHEYAEMASRTESMSGQMSEDLSQQFAQYVMKNAPQDVEAILTNTSSP<br>EIAERRRAMAWSFVQEQVQPGVDNTWRESRRDIGKGMESVPSGGGSQDIIADHQGHQAIIEQRTQD<br>SNIRNDVKHQVDNMVTEYRGNIGDTQNSIRGEENIVKGQYSELQNHHKTEALTQNNKYNEEKLAQ<br>ERIPGADSPKELLEKAKSYQHKE
TraU	330	26%	BAA97957	P18471	MKRRLWLLMLFLFAGHVPAASADSACEGRFVNPITDICWSCIFPLSLGSIKVSQGKVPDTANPSMPI<br>QICPAPPPLFRRIGLAIGYWEPMALTDVTRSPGCMVNLGFSLPAFGKTAQGTAKKDEKQVNGAFYH<br>VHWYKYPLTYWLNIITSLGCLEGGDMDIAYLSEIDPTWTDSSLTTILNPEAVIFANPIAQGACAADAI<br>ASAFNMPLDVLFWCAGSQGSMYPFNGWVSNESSPLQSSLLVSERMAFKLHRQGMIMETIGKNNAV<br>CNEYPSPILPKERWRYQMVNMYPDSGQCHPFGRSVTRWETGKNPPNTKKNFGYLMWRKRNCVFL

TraI	1756	20%	BAA97974	P14565	MMSIAQVRSAGSAGNYYTDKDNYYVLGSMGERWAGRGAEQLGLQGSVDKDVFTRLLEGRLPDG<br>ADLSRMQDGSNRHRPGYDLTFSAPKSVSMMAMLGGDKRLIDAHNQAVDFAVRQVEALASTRVM<br>TDGQSETVLTGNLVMALFNHDTSRDQEPQLHTHAVVANVTQHNGEWKTLSSDKVGKTGFIENVY<br>ANQIAFGRLYREKLKEQVEALGYETEVVGKHGMWEMPGVPVEAFSGRSQTIREAVGEDASLKSRD<br>VAALDTRKSKQHVDPEIKMAEWMQTLKETGFDIRAYRDAADQRADLRTLTPGPASQDGPDVQQA<br>VTQAIAGLSERKVQFTYTDVLARTVGILPPENGVIERARAGIDEAISREQLIPLDREKGLFTSGIHVLD<br>ELSVRALSRDIMKQNRVTVHPEKSVPRTAGYSDAVSVLAQDRPSLAIVSGQGGAAGQRERVAELV<br>MMAREQGREVQIIAADRRSQMNMKQDERLSGELITGRRQLLEGMAFTPGSTVIVDQGEKLSLKET<br>LTLLDGAARHNVQVLITDSGQRTGTGSALMAMKDAGVNTYRWQGGEQRPATIISEPDRNVRYAR<br>LAGDFAASVKAGEESVAQVSGVREQAILTQAIRSELKTQGVLGLPEVTMTALSPVWLDSRSRYLRD<br>MYRPGMVMEQWNPETRSHDRYVIDRVTAQSHSLTLRDAQGETQVVRISSLDSSWSLFRPEKMPVA<br>DGERLRVTGKIPGLRVSGGDRLQVASVSEDAMTVVVPGRAEPATLPVSDSPFTALKLENGWVETP<br>GHSVSDSATVFASVTQMAMDNATLNGLARSGRDVRLYSSLDETRTAEKLARHPSFTVVSEQIKTR<br>AGETSLETAISHQKSALHTPAQQAIHLALPVVESKKLAFSMVDLLTEAKSFAAEGTGFTELGGEINA<br>QIKRGDLLYVDVAKGYGTGLLVSRASYEAEKSILRHILEGKEAVMPLMERVPGELMEKLTSGQRA<br>ATRMILETSDRFTVVQGYAGVGKTTQFRAVMSAVNMLPESERPRVVGLGPTHRAVGEMRSAGVD<br>AQTLASFLHDTQLQQRSGETPDFSNTLFLLDESSMVGNTDMARAYALIAAGGGRAVASGDTDQLQ<br>AIAPGQPFRLQQTRSAADVAIMKEIVRQTPELREAVYSLINRDVERALSGLESVKPSQVPRQEGAW<br>APEHSVTEFSHSQEAKLAEAQQKAMLKGEAFPDVPMTLYEAIVRDYTGRTPEAREQTLIVTHLNED<br>RRVLNSMIHDVREKAGELGKEQVMVPVLNTANIRDGELRRLSTWETHRDALVLVDNVYHRIAGIS<br>KDDGLITLQDAEGNTRLISPREAVAEGVTLYTPDTIRVGTGDRMRFTKSDRERGYVANSVWTVTA<br>VSGDSVTLSDGQQTREIRPGQEQAEQHIDLAYAITAHGAQGASETFAIALEGTEGNRKLMAGFESA<br>YVALSRMKQHVQVYTDNRQGWTDAINNAVQKGTAHDVFEPKPDREVMNAERLFSTARELRDVA<br>AGRAVLRQAGLAGGDSPARFIAPGRKYPQPYVALPAFDRNGKSAGIWLNPLTTDDGNGLRGFSGE<br>GRVKGSGDAQFVALQGSRNGESLLADNMQDGVRIARDNPDSGVVVRIAGEGRPWNPGAITGGRV<br>WGDIPDNSVQPGAGNGEPVTAEVLAQRQAEEAIRRETERRADEIVRKMAENKPDLPDGKTEQAVR<br>EIAGQERDRAAITEREAALPEGVLREPQRVREAVREIARENLLQERLQQMERDMVRDLQKEKTLG<br>GD
TraY	131	25%	BAA97943	P06627	MKRFGTRSATGKMVKLKLPVDVESLLIEASNRSGRSRSFEAVIRLKDHLHRYPKFNRAGNIYGKSL<br>VKYLTMRLDDETNQLLIAAKNRSGWCKTDEAADRVIDHLIKFPDFYNSEIFREADKEEDITFNTL
TraM	127	33%	BAA97941	P10026	MAKVNLYISNDAYEKINAIIEKRRQEGAREKDVSFSATASMLLELGLRVHEAQMERKESAFNQTEF<br>NKLLLECVVKTQSSVAKILGIESLSPHVSGNSKFEYANMVEDIREKVSSEMERFFPKNDDE

TraD	717	29%	BAA97972	P09130	MSFNAKDMTQGGQIASMRIRMFSQIANIMLYCLFIFFWILVGLVLWIKISWQTFVNGCIYWWCTTL<br>EGMRDLIKSQPVYEIQYYGKTFRMNAAQVLHDKYMIWCSEQLWSAFVLAAVVALVICLITFFVVS<br>WILGRQGKQQSENEVTGGRQLTDNPKDVARMLKKDGKDSDIRIGDLPIIRDSEIQNFCLHGTVGAG<br>KSEVIRRLANYARQRGDMVVIYDRSGEFVKSYYDPSIDKILNPLDARCAAWDLWKECLTQPDFDN<br>TANTLIPMGTKEDPFWQGSGRTIFAEAAYLMRNDPNRSYSKLVDTLLSIKIEKLRTYLRNSPAANLV<br>EEKIEKTAISIRAVLTNYVKAIRYLQGIEHNGEPFTIRDWMRGVREDQKNGWLFISSNADTHASLKP<br>VISMWLSIAIRGLLAMGENRNRRVWFFCDELPTLHKLPDLVEILPEARKFGGCYVFGIQSYAQLEDI<br>YGEKAAASLFDVMNTRAFFRSPSHKIAEFAAGEIGEKEHLKASEQYSYGADPVRDGVSTGKDMER<br>QTLVSYSDIQSLPDLTCYVTLPGPYPAVKLSLKYQTRPKVAPEFIPRDINPEMENRLSAVLAAREAE<br>GRQMASLFEPDVPEVVSGEDVTQAEQPQQPVSPAINDKKSDSGVNVPAGGIEQELKMKPEEEMEQ<br>QLPPGISESGEVVDMAAYEAWQQENHPDIQQQMQRREEVNINVHRERGEDVEPGDDF
TraT	244	34%	BAA97971	P13979	MMKTKKLMMVALVSSTLALSGCGAMSTAIKKRNLEVKTQMSETIWLEPASERTVFLQIKNTSDKD<br>MSGLQGKIADAVKAKGYQVVTSPDKAYYWIQANVLKADKMDLRESQGWLNRGYEGAAVGAAL<br>GAGITGYNSNSAGATLGVGLAAGLVGMAADAMVEDVNYTMITDVQIAERTKATVTTDNVAALR<br>QGTSGAKIQTSTETGNQHKYQTRVVSNANKVNLKFEEAKPVLEDQLAKSIANIL
TraS	173	8%	BAA97970	P09129	MITQQIISSELEVLKKHIDSGDIRIPSLWQGLKPGLIIMGWMIFCPLLMSFLITQKTSETLTAVLAGGW<br>LGLIILFIVARIRMLYFSLPEEFLKTSSVMRVISSKLKVYFIVYMGVIFLWSFLGGGIIYGFGAILVTVI<br>MAFLIQLDIGRYQFVGVIDAINSYVKNKKLSRVK

*Disordered sequences of transfer proteins were predicted using the Phyre2 algorithm [1], using the amino acid sequences of the transfer proteins in FASTA format as input via the Phyre2 web interface. Results were manually collated from Phyre2 outputs, and regions of predicted disorder are shaded in grey in the amino acid sequences above.

References

1. Kelley, L.A.; Sternberg, M.J.E. Protein structure prediction on the Web: a case study using the Phyre server. *Nat. Protoc.* **2009**, *4*, 363–371, doi:10.1038/nprot.2009.2.
